# Cell fusion enhances energy metabolism of mesenchymal tumor hybrid cells to sustain their proliferation and invasion

**DOI:** 10.1186/s12885-021-08561-6

**Published:** 2021-07-28

**Authors:** Ariadna Brito, Candice Merle, Pauline Lagarde, Benjamin Faustin, Anne Devin, Lydia Lartigue, Frederic Chibon

**Affiliations:** 1 Cancer Research Center in Toulouse (CRCT), INSERM U1037, 31037 Toulouse, France; 2 University of Toulouse 3, Paul Sabatier, 118 route Narbonne, 31062 Cedex 9 Toulouse, France; 3 INSERM U1218, 299 cours de l’Argonne, F-33076 Bordeaux, France; 4 University of Bordeaux, 146 rue Léo Saignat, F-33000 Bordeaux, France; 5 Department of Biopathology, Bergonie Institute, 229 cours de l’Argonne, F-33076 Bordeaux, France; 6 CNRS UMR 5164, 33000 Bordeaux, France; 7 Immunology Discovery, Janssen Research and Development, San Diego, CA USA; 8 CNRS UMR 5095, 1 Rue Camille Saint-Saëns, F-33077 Bordeaux Cedex, France; 9 Department of Pathology, Institut Claudius Régaud, IUCT-Oncopole, Toulouse, France

**Keywords:** Cell fusion, Energy metabolism, AICAR, AMPK, Invasion

## Abstract

**Background:**

Cell-to-cell fusion is emerging as a key element of the metastatic process in various cancer types. We recently showed that hybrids made from the spontaneous merging of pre-malignant (IMR90 E6E7, i.e. E6E7) and malignant (IMR90 E6E7 RST, i.e. RST) mesenchymal cells recapitulate the main features of human undifferentiated pleomorphic sarcoma (UPS), with a highly rearranged genome and increased spreading capacities. To better characterize the intrinsic properties of these hybrids, we investigated here their metabolic energy profile compared to their parents.

**Results:**

Our results unveiled that hybrids harbored a Warburg-like metabolism, like their RST counterparts. However, hybrids displayed a much greater metabolic activity, enhancing glycolysis to proliferate. Interestingly, modifying the metabolic environmental conditions through the use of 5-aminoimidazole-4-carbox-amide-1-β-D-ribofuranoside (AICAR), an activator of the 5′-adenosine monophosphate (AMP)-activated protein kinase (AMPK), specifically reduced the growth of hybrids, and also abrogated the invasive capacity of hybrids displaying enhanced glycolysis. Furthermore, AICAR efficiently blocked the tumoral features related to the aggressiveness of human UPS cell lines.

**Conclusion:**

Altogether, our findings strongly suggest that hybrids rely on higher energy flux to proliferate and that a drug altering this metabolic equilibrium could impair their survival and be potentially considered as a novel therapeutic strategy.

**Supplementary Information:**

The online version contains supplementary material available at 10.1186/s12885-021-08561-6.

## Introduction

Cell fusion is a normal physiological event that plays a critical role in fertilization, placentation, myogenesis, osteogenesis, wound healing and tissue regeneration [1–4]. In addition, it is strongly suggested to be a tumor inception contributor, progression and heterogeneity [5]. Several studies have demonstrated the presence of hybrid cells in human cancers, in some cases comprising up to 40% of tumors [6]. Recently, *Gast* et al. have shown that hybrid cells could be found in both human pancreatic ductal adenocarcinoma cells and in the circulatory system, where they were associated with a poor prognosis [7]. Moreover, our team recently reported that the fusion of pre-malignant and malignant mesenchymal cells triggers a genomic instability that resembles the instability found in human sarcomas [8], and also showed that hybrids made from cancer mesenchymal cells with non-cancerous partners gain invasiveness, giving rise to highly metastatic tumors [8, 9].

Hybrids resulting from homotypic or heterotypic cancer cells fusion are known to exhibit features such as high aggressiveness, drug resistance, metastatic capabilities, and to facilitate tumor proliferation when compared to non-hybrid parental cells [6–9]. While hybrids can inherit genetic, and thus phenotypic, features from their parents, they also develop their own identity as a result of the intense remodeling of their genome, epigenome and transcriptome [10, 11]. However, it is still unclear how these major cellular changes drive the gain-of-functions observed in hybrid cells and which molecular mechanisms are specifically activated upon fusion to allow greater dissemination and/or growth abilities.

Like polyploid cells, hybrids host a higher DNA content and are larger in size than their euploid counterparts [12]. This greater cell volume and chromosome number presumes higher metabolic needs to ensure the continuity of cell division, growth and all basic cellular functions [13]. For example, hyperploid glioblastoma cells were shown to be more metabolically active than their euploid peers [13]. Along with immune evasion, metabolic reprograming is now one of the main hallmarks of cancer cells, and cellular energetics are considered as core traits of tumors, playing a major role in cancer cell proliferation and metastatic spreading [14, 15].

Cancer cells generally use glycolysis instead of oxidative phosphorylation (OXPHOS) despite having enough oxygen levels. This phenomenon, known as the “Warburg Effect” [16], confers an advantage by increasing the level of non-oxidative ATP and generating intermediates that are important for cell growth and dissemination [17, 18]. 5′-adenosine monophosphate (AMP)-activated protein kinase (AMPK) is a master sensor of cellular energy and adaptor to metabolic stress in cancer cells [19]. Previous studies have established that AMPK is related in a complex manner with other metabolic/energy pathways such as SIRT1, Akt, mTOR and PARPs [19–21]. AMPK is generally downregulated in cancer cells, thereby favoring ATP-consuming mechanisms including proliferation [22]. The activation of AMPK has been shown to induce mitochondrial biogenesis triggering an anti-Warburg [23] and anti-proliferative [21] effect in several types of cancer [24].

Modifying the intracellular energy metabolism is thus critical for the growth and dissemination of cancer cells. While many reports agree that hybrids display greater pro-oncogenic potential, little is known regarding their metabolic status or the metabolic consequences of cell fusion. It is highly possible that, upon merging, hybrids undergo extensive metabolic rewiring and must adapt to find the right equilibrium to primarily sustain their needs and pursue their development. Nonetheless, it is also possible that these metabolic changes trigger a higher metabolic stress, while supporting the acquisition of novel functions.

We recently showed that cell fusion promotes tumor progression (notably metastatic spread) by using hybrids made from the spontaneous cell fusion of IMR90 E6E7 RST (malignant) and IMR90 E6E7 (pre-malignant) cells [9]. In the present study we used this well characterized model of mesenchymal tumor progression to investigate changes in energy metabolism operating in hybrid cells, following the hypothesis that their aggressiveness may result from an increased metabolic stress altering the AMPK pathway. To address these questions, we measured their mitochondrial respiration, glycolytic activity, and evaluated the effect of AMPK activation on their growth and motility.

## Results

### Hybrids display enhanced energy metabolic expression profiles

**Fig. 1:**
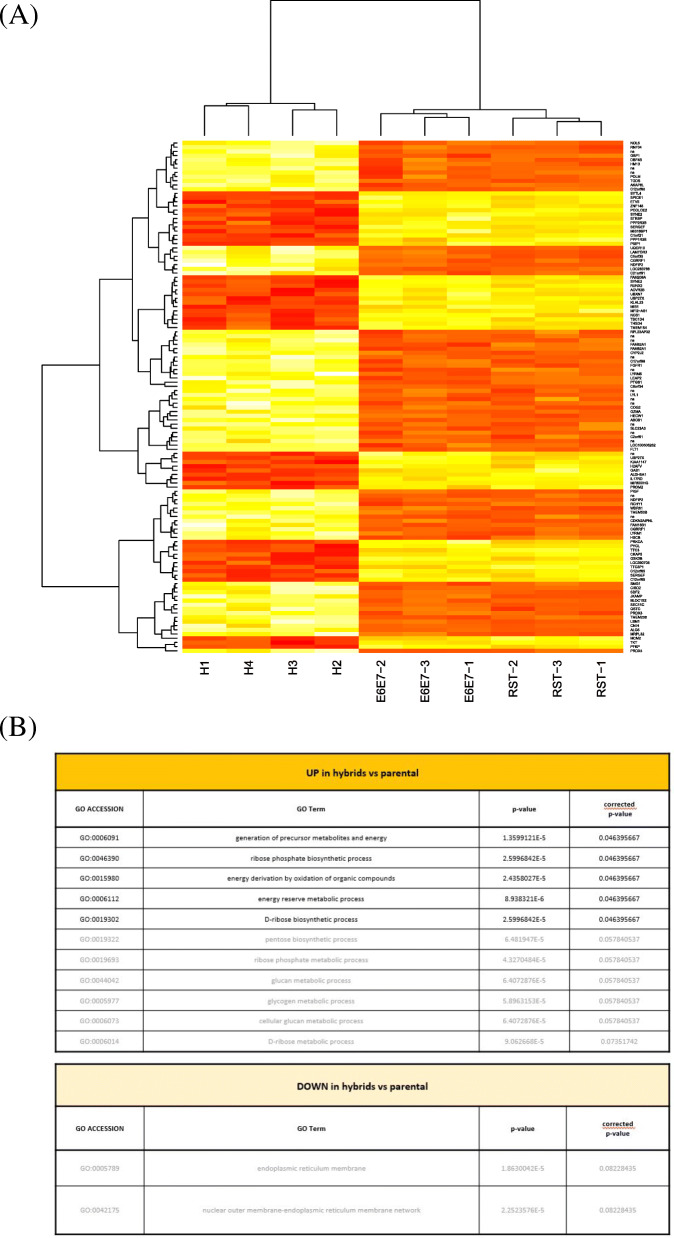
Genes differentially expressed in hybrids compared to parental cells. **A** Heatmap with the 122 significantly (p (corr) < 0,005) differentially expressed genes between parental (RST and E6E7) and hybrid cells (see supplementary Table 1). **B** GO obtained after analysis of the genes the most upregulated in hybrids vs. parents (upper panel) and the most downregulated in hybrids vs. parents (lower panel)

To elucidate the origin of the increased aggressiveness of hybrids, we performed gene expression profiling on E6E7, RST and four hybrids (H1-H4, Supplementary Fig. 1A), and analyzed the gene ontology (GO) of the 122 genes which were the most differentially expressed in hybrids compared to their parental cells (Fig. 1, A and B, and supplementary Tables 1 and 2). No significant enriched pathways emerged from the GO analysis of the most downregulated genes in hybrids (Fig. 1B, lower panel). However, five GOs related to metabolic processes, especially energy metabolism was found to be significantly up-regulated in hybrids compared to their parents (Fig. 1B, upper panel), indicating that hybrids required higher energy rates than their euploid counterparts. To explore experimentally the significance of these data, we then performed respiration and glucose consumption measurements on hybrids and their parents.

### Hybrid cells display respiratory rates equivalent to RST cells

**Fig. 2:**
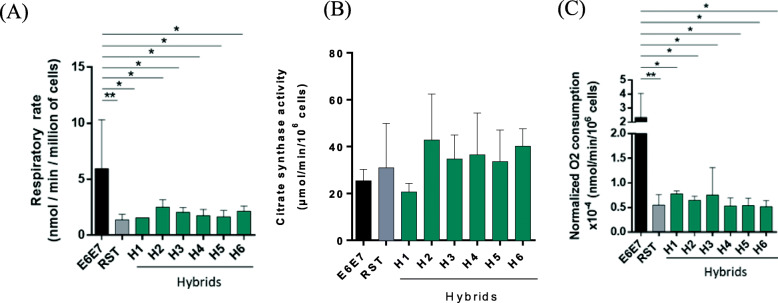
IMR90 E6E7/E6E7 RST hybrid cells exhibit basal respiratory rates similar to RST parental cells. **A** Respiratory rate of E6E7, RST and H1-H6 hybrids under pyruvate. Values normalized to number of cells and expressed in nmol of O_2_ consumed per minute for one million cells (*n* = 2 for H1, H5; *n* = 3 for E6E7, H2, H3, H4, H6; *n* = 6 for RST; each experiment done in duplicate). Statistical analyses were done using one-way ANOVA test followed by Holm-Sidak multiple comparison test (*p < 0.05, **p < 0.01; error bars, SD). **B** Citrate synthase activity was evaluated for two parental and all hybrid cells directly from samples used to determine respiration rate as described in (**a**). Statistical analyses were done using one-way ANOVA test followed by Holm-Sidak multiple comparison test (error bars, SD). **C** Citrate synthase normalized respiratory rate of E6E7, RST and H1-H6 hybrids under pyruvate. Each value obtained in (**A**) was normalized by citrate synthase activity found in the same sample. Statistical analyses were done using one-way ANOVA test followed by Holm-Sidak multiple comparison test (*p < 0.05, **p < 0.01; error bars, SD)

To determine the energy metabolism of hybrids, pre-malignant E6E7 and malignant RST cells, we first measured basal respiration in intact cells under pyruvate (VO_2 pyr_) (Fig. 2). As expected, E6E7 cells displayed a higher respiratory rate than transformed RST cells, both before (Fig. 2A) and after (Fig. 2C) normalization of the data with citrate synthase activity, used here as a validated enzyme marker of mitochondrial mass (Fig. 2B and C). Interestingly, all hybrids exhibited basal oxygen consumption rates similar to RST cells, i.e. lower than the non-fully transformed E6E7 cells.

Additionally, we showed that RST and hybrids respiratory rates remained lower than E6E7 when respiration was blocked with oligomycin to assess the non-phosphorylating respiratory rate (VO_2 oligo_, Supplementary 2A and B, upper left panels). Maximal respiration (VO_2_, DNP) showed that H3 (alike E6E7 cells) may have greater mitochondrial respiratory chain capacities (Supplementary Fig. 2, A and B, upper right panels), and may use less of its mitochondrial capacity (10%) than the other cell lines (between 20 to 40%) (Supplementary Fig. 2, A and B, lower right panels). Finally, the VO_2 pyr_/VO_2 oligo_ ratio revealed no major differences between the various cell lines, indicating that the mitochondrial ATP synthesis was equivalent in all samples (Supplementary Fig. 2, A and B, lower left panels). Altogether, these results showed that hybrid cells have a respiration rate profile similar to that of the transformed RST cells.

### Hybrid cells up-regulate glycolysis

**Fig. 3:**
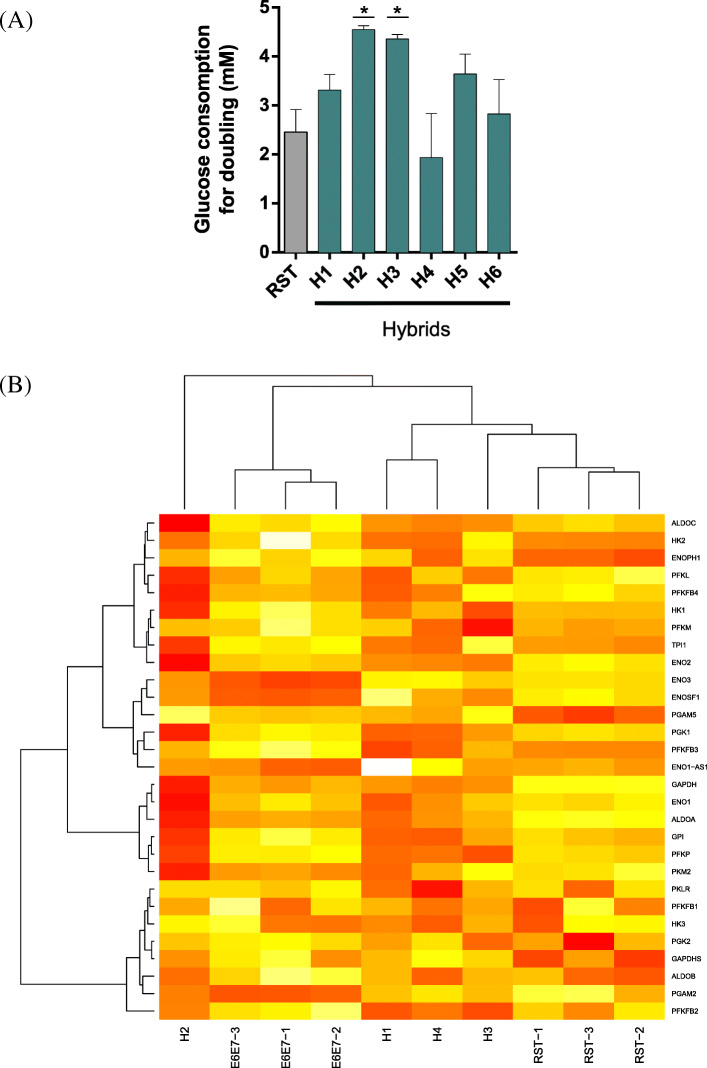
IMR90 E6E7/E6E7 RST hybrid cells are more metabolically active than RST cells. **A** Average glucose consumption of parental and hybrids cells per doubling time (*n* = 2 in duplicate; error bars, SD). Data were normalized to the growth rate of each cell line established in parallel of the experiments, as shown in Supplemental Fig. 3b. **B** Clustering of the nine samples with 29 genes involved in glycolysis

In addition to oxygen consumption, we compared the rate of glucose consumption between the parental and the hybrid cells (i.e. energy production through glycolysis). As expected, transformed RST cells displayed high rates of glucose consumption (Supplementary Fig. 3A), contrasting with immortalized E6E7 cells. All hybrids showed intermediate rates. However, to be able to compare the samples, we then calculated the quantity of glucose used produced per doubling time. Doubling times were determined from growth curves established in parallel of glucose measurements (Supplementary Figure 3B and [9]). E6E7, which barely proliferates (doubling time > 6 days), was not included in this analysis. As shown in Fig. 3A, hybrids used at least as much glucose as RST cells to double. Interestingly, five out of six hybrids consumed higher amount of glucose than the parental RST to double; H2 and H3 showing a very high increase (> 80%) while H1, H5 and H6 a milder rise (comprised between 20 and 50%). Only H4 did not use higher glucose amount than RST. To sum up, hybrid cells displayed a Warburg-like metabolism like their oncogenic RST parental cells. Moreover, hybrids were likely to be metabolically more active than RST parental cells, with a strong tendency to enhance glycolysis (5 out 6 clones).

This tendency was confirmed when we analyzed the RNA expression level for 29 genes related to glycolysis both in hybrids and parents (Fig. 3B) using the gene expression data obtained in section 2.1. Interestingly, hybrids not only clustered separately from E6E7 and RST samples, but they also displayed higher expression level of multiple genes of the glycolytic pathway. H2 exhibited the highest increase, and H1, H3 and H4 clustered together closer to RST.

### AICAR impairs proliferation and invasive properties of hybrids displaying lower AMPK (alpha 1) expression level

**Fig. 4:**
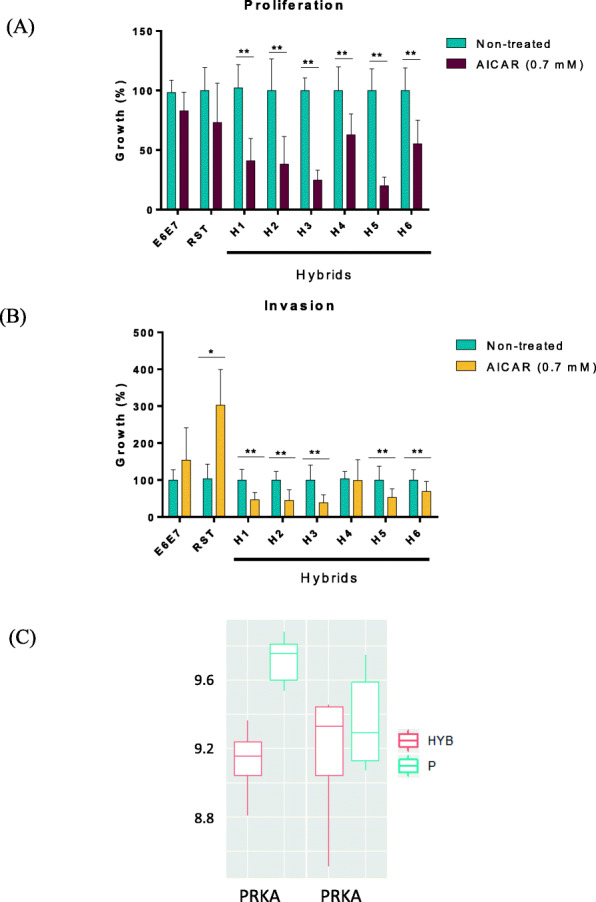
AICAR significantly reduces proliferative and invasive capacities of hybrids overexpressing glycolysis genes and down regulate AMPK. **A** Growth of E6E7, RST and H1-H6 hybrid cells in absence or presence of AICAR treatment (700 μM for 6 days) evaluated by flow cytometry. For each cell line, data are expressed as growth percentage compared to the growth of the corresponding untreated sample (100%). Statistical analyses were performed using a t-test (nonparametric) Mann-Whitney (**p < 0.01, error bars, SD; *n* = 6 in triplicates). **B** Invasion of E6E7, RST and H1-H6 hybrid cells post-vehicle (H_2_O) or AICAR treatment (700 μM for 24 h of pre-treatment) assessed using Matrigel-coated Boyden chambers. Invasion evaluated at T = 18 h. Data were normalized to the number of cells seeded at T0 determined by flow cytometry. Statistical analyses were done using a t-test (nonparametric) Mann-Whitney (*p < 0.05, **p < 0.01, error bars, SD; *n* = 5 in quadruplicate in each independent experiment). **C** Comparison of the AMPK expression genes, i.e. PKRKAA1 and PRKAB1 (Wilcoxon rank sum test, *p* = 0.009524). P corresponds to E6E7 and RST samples altogether and HYB corresponds to H1 to H4 altogether

Data presented above indicate that hybrids display a Warburg-energetic metabolism and seem to rely on high glycolytic flux. To investigate the importance of glycolysis in hybrids, we then treated the cells with AICAR (a direct activator of the AMPK kinase [20]) known to enhance mitochondrial biogenesis and to promote oxidative phosphorylation (among others mechanisms) [19, 23, 25]. At 700 μM, AICAR drastically and strictly reduced cell proliferation in all the hybrids (Fig. 4A), implying that re-activating AMPK abrogated their cell cycling and expansion, whereas it had no significant effect on the proliferation of E6E7 and RST parental cells [9]. Furthermore, we previously reported that H1-H6 hybrids display greater invasive capacities than RST cells (except H4 in vitro) [9]. Therefore, we investigated the effect of AICAR on cell invasion using a Matrigel-coated Boyden chamber (Fig. 4B). Interestingly, all hybrids except H4 showed significant sensitivity to AICAR, being barely able to cross the coated membrane, while RST cells showed the opposite. Altogether, these results likely imply that blocking the metabolic changes occurring in hybrids using AICAR impairs their growth and potentially their invasive properties, offering a potential therapeutic window to target these harmful cells.

Given the effect of AICAR, we then evaluated the effect of other pharmacological agents targeting the intracellular metabolism. Surprisingly, we observed that enhancing mitochondrial and OXPHOS metabolism using another method, i.e. switching glucose to galactose in culture medium (as previously described [26],) blocked both the growth of parental RST and hybrids (Supplementary Fig. 4A), thus showing no exclusive activity against hybrids, contrary to AICAR. E6E7 was resistant to this switch, presumably because it has a higher OXPHOS metabolism (Fig. 2). Preliminary data also indicated no hybrid-specific effect of (i) glutamine removal that limits glutaminolysis (Supplementary Fig. 4B), (ii) 2-deoxyglucose, an inhibitor of glycolysis that equally prevented the growth of both parents and hybrids (not shown) and (iii) metformin, an agent targeting AMPK via mitochondrial respiratory chain complex I, i.e. another mechanism than AICAR [20] that impaired both RST and hybrids growth (not shown).

To deeper understand the specific action of AICAR against hybrids, we then checked the RNA expression level of one of its well-known targets i.e. AMPK, using our microarray data (Fig. 4C). Interestingly, while AMPK subunit-beta-1 (PRKAB1; non-catalytic) expression level was similar in all samples, AMPK subunit -alpha-1 (PRKAA1; catalytic) was significantly under-expressed in hybrids (H1-H4 taken together) compared to their parents (E6E7 and RST samples altogether). This result was also evaluated by western blot using an antibody against AMPK α1/α2 isoforms against the same samples employed in the microarray experiment (Supplementary Fig. 5A, B). A higher variability of AMPK expression was observed in the samples possibly due to the lack of specificity of the antibody against the α1 isoform of AMPK (i.e. H1 displayed the lowest expression compared to its parents; H2 was close to E6E7, and H3 in-between E6E7 and RST samples. H4 did not seem to express lower levels of AMPK α1/α2).

### Pleomorphic sarcoma cells are sensitive to AICAR

**Fig. 5:**
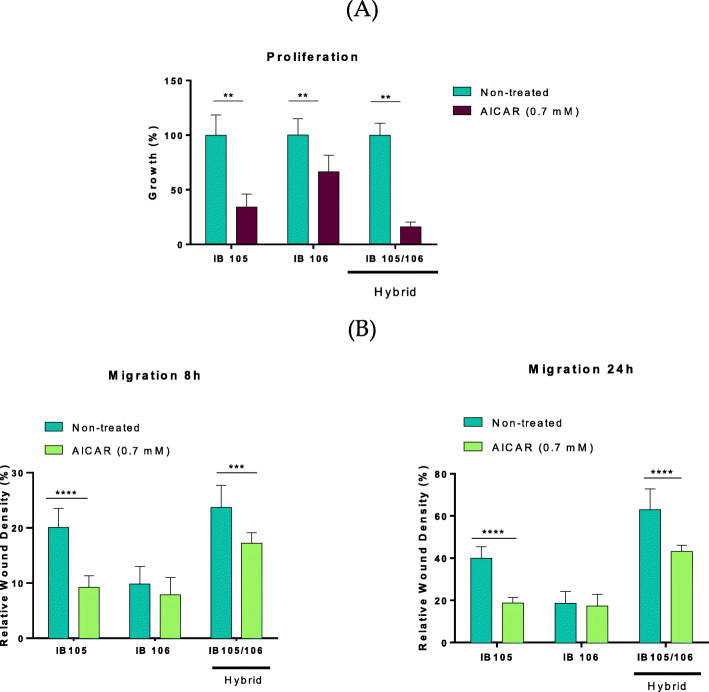
Aneuploid pleomorphic sarcoma cells are sensitive to AICAR treatment. **A** Growth of IB105 DsRed, IB106 GFP and IB105/106 hybrids in absence or presence of AICAR treatment (700 μM for 6 days) evaluated by flow cytometry. For each cell line, data are expressed as growth percentage compared to the growth of the untreated sample (100%). Statistical tests were done using a t-test (nonparametric) Mann-Whitney (**p < 0.01, error bars, SD; *n* = 5 done in sextuplicates twice and in triplicates for the rest of the experiments). **B** Migration of IB105 DsRed, IB106 GFP and IB105/106 cells post-vehicle or AICAR pre-treatment (700 μM for 24 h) evaluated by wound healing assays using IncucyteS3. Data represent relative wound density at 8 or 24 h post-scratch. Statistics were done using a t-test. (Nonparametric) Mann-Whitney (***p < 0.001, ****p < 0.0001, error bars, SD; *n* = 2 done in 12 wells for each sample, condition and per experiment)

We previously reported that H1-H6 hybrids develop undifferentiated pleomorphic sarcoma (UPS) very close to human UPS when engrafted into mice [9]. UPS are highly aggressive aneuploid cells that are prone to cell fusion events [9]. Following the hypothesis that UPS development could emerge from (or could include) cell fusion events, we tested the effect of AICAR on these tumors. Two cell lines derived from a single human UPS were generated and named IB105 DsRed and IB106 GFP respectively. These two cell lines (genetically and phenotypically different) were co-cultured and hybrids were selected to obtain a pure hybrid sarcoma cell line (i.e. IB105/106) (Supplementary Fig. 1B, and [9]). All three cell lines treated with AICAR showed a high sensitivity to the drug in proliferation assays (Fig. 5A). Despite several attempts, these cell lines exhibited insufficient in vitro invasive capacity to be challenged with AICAR. We thus monitored their migration abilities using wound healing assays in the presence and absence of the compound (Fig. 5B right and left panels). From growth curves published in [9] the doubling time for IB105, IB106 and IB105/106 is 71.8, 56.06 and 63.5 h, respectively, and, therefore, should not interfere with the migration assays. Interestingly, AICAR annihilated both the mobility of IB105 and IB105/106 (Fig. 5B, supplementary Fig. 6, A and B) (IB106 being less motile).

## Discussion

Cell fusion can lead to the production of highly malignant harmful cells at various steps of the oncogenic process [27, 28], representing a potential target for therapeutic intervention. However, to reach that goal, it is essential to characterize the biological changes occurring in hybrid cells compared to their parents and to decipher the signaling pathways that are crucial for their survival and growth. Until now, hybrids have been shown to undergo a complex reorganization of their genomic, epigenomic and transcriptomic content [29], but no link has been established between these events and their higher pro-oncogenic functions.

To better characterize hybrid cells and formulate new hypothesis regarding their increased malignity, we performed gene expression profiling experiments using four metastatic hybrid cell lines (H1-H4) and their non-metastatic parental cells (i.e. pre-malignant E6E7 and malignant RST) [9]. Interestingly, GO analysis of the most upregulated genes in hybrids vs. parents evidenced an enrichment in the pathways related to metabolism, especially energy metabolism. We thus explored the idea that, concurrently to their nuclear reprogramming, hybrid cells might undergo a remodeling of their energy traits to secure their growth and sustain their higher invasiveness.

In agreement with a higher metabolic imbalance in hybrids, we found that AICAR, a pharmacological agent targeting AMPK which is a major energy sensor in the cell, specifically blocked the proliferation of hybrids while having no effect on RST and E6E7 parental cells. In addition, AICAR attenuated the invasive processes in hybrids with enhanced glycolysis (i.e. H1, H2, H3, H5 and H6). As described above, H4 did not show evidence of glycolysis hyper-activation compared to RST and, hence, it is possible that AICAR only precludes, (or is most effective on) the invasive processes driven by glycolysis.

Altogether, our results highlight the anti-proliferative effect of AICAR on hybrid cells using cell counting by flow cytometry. This assay selects the cells based on their physical properties (size, granularity), and presents the advantage to be simple and straightforward when manipulating poorly characterized or complex cells (hybrids vs. non-hybrids).

Surprisingly, AICAR enhanced the invasive capacities of parental RST cells, perhaps due to the higher expression level of AMPK in these cells compared to hybrid cells. Previous studies have shown that AMPK can function as a tumor promotor under certain circumstances, notably when *PRKAA1,* coding the catalytic subunit-alpha-1 of AMPK, is highly expressed [30]. Moreover, it is important to consider that RST cells generate UPS-like tumors in vivo that lack many features of the corresponding human malignant tumors (i.e. no genetic instability and no metastatic capacities), contrary to hybrids (i.e. highly aneuploid and metastatic) [9]. In that sense, RST cells do not really mirror the cancer cells found in human UPS whereas hybrid cells do, therefore identifying drugs able to disrupt the propagation of hybrids was the ultimate goal. Altogether, these results emphasize the value of treating hybrid cells with AICAR as previously reported for other aneuploid cancer cells [31].

Interestingly, transcriptome analysis of parental and hybrid cells further revealed a specific decrease in the expression of the catalytic subunit-alpha-1 of AMPK (*PRKAA1*) in hybrid cells and no modification of the regulatory subunit (*PRKAB1*). Western blot confirmed a tendency of a decrease of AMPK alpha in hybrids with the limitation that the antibody was directed against alpha1 and alpha2. isoforms, and not solely against alpha1. AMPK is a major mediator of cellular energy homeostasis and its loss might thus reflect a selection pressure occurring specifically in hybrids, which presumably require more energy to survive. Indeed, analysis of the energy metabolic profiles of hybrid and parental cells (respiration rates and glucose consumption) revealed hybrids to be inclined to higher metabolic activities. Not only did they rely on a ‘Warburg-like’ metabolism like RST malignant cells (i.e. lower respiratory rate and higher glucose consumption), but also on greater metabolic flux. Indeed, five hybrids out six (i.e. H1, H2, H3, H5 and H6) used more glucose (significantly for H2 and H3) than RST cells, underlining their greater glycolytic rates.

Such increases might be necessary to mobilize sufficient amounts of nutrients and energy for anabolic reactions and growth [32]. In this study, only H4 did not show higher glycolytic requirements. It still remains possible that this particular hybrid relies on other critical metabolic pathways for growth, such as glutamine metabolism [33], but this would need further investigation. Moreover, corroborating these results, we found that hybrids up-regulated several genes of the glycolytic pathway at the mRNA level. Remarkably, many genes of glycolysis have been previously implicated in cancer progression [34]. To sum up, our results revealed that hybrids are more metabolically active than their RST euploid counterpart, relying essentially on higher glycolytic rates (but also possibly on other pathways) to survive and proliferate. Interestingly, overactivating glycolysis leads to the production of lactate, an oncometabolite well known for its role in immune evasion and metastatic development, a characteristic recurrently found in H1 to H6 hybrid cells [9, 34–37,38].

The activation of AMPK was previously shown to trigger mitochondrial biogenesis and OXPHOS, causing anti-Warburg [23] and anti-proliferative [21] effects in several types of cancer [24], such as leukemia [39], breast cancer [40], pancreatic cancer [41], hepatocellular carcinoma [42], and prostate cancer [43]. Here, we show that AICAR, a direct AMPK activator, is a potent agent against aneuploid hybrid cells functioning at high metabolic rates. Interfering with the metabolic status of hybrids (using AICAR here) seems to revoke both their growth and invasive capacity. Thus, it is likely that the over-activated metabolism of hybrids is necessary for their proliferation and invasion properties, creating an interesting therapeutic target. It is to note, that our first attempts to use metformin, another potential activator of AMPK did not show any selectivity against hybrids. However, AMPK is only an indirect target of metformin [20], which can explain this result. As described above, we recently proved that cell fusion of E6E7 and RST cells creates hybrids able to generate UPS-like tumors with all the clinical traits of their human counterparts [8, 9]. Suspecting that human UPS cells may be the result of cell fusion events, we then attempted to treat them with AICAR. Interestingly, IB105, IB106 and the fusion IB105/106 cells were all highly sensitive to AICAR treatment, exhibiting a drastic decrease in proliferation and migration (except for IB106 that barely migrates) in presence of the drug.

Altogether, our findings show that cell fusion generates hybrid cells with greater metabolic needs compared to their parents and that this specific feature may represent their Achilles heel, paving the way for new therapeutic approaches to treat human pleomorphic sarcoma and eradicate these aggressive cancer cells.

## Materials and methods

### Cell lines and reagents

IB105, IB106 and IB105/106 sarcoma cell lines, as well as all the IMR90 fibroblasts (1/IMR90 E6E7 CFP Blast^R^ (i.e. E6E7), and 2/IMR90 E6E7 HRAS_G12V_ SmallT Tert DsRed Puro^R^ (i.e. RST)) were cultured in RPMI-1640 (Gibco, Invitrogen) supplemented with 10% fetal bovine serum (FBS) at 37 °C in a humidified CO_2_ incubator. Sarcoma cells were generated in the laboratory [44], while IMR90 cell lines were kindly given by M. Teichmann [45]. The latter were generated according to the cell transformation model described by Hahn et al. [46, 47]. AICAR was purchased from Sigma Aldrich (Ref. A9978, St. Luis, MO).

### Hybrid cells selection

As previously published in [9], DsRed, GFP and CFP parental cell lines were generated by lentiviral infection using pSD136-puromycin-DsRed, pER69-puromycin-GFP, pER167-Blasticidin-CFP plasmids, respectively. 150,000 cells of each parental cell line were seeded together in 6-well plates. Spontaneous hybrid cells were selected after 72 h of contact by cell sorting (IB105/106) or double antibiotic addition (puromycin/blasticidin for IMR90 E6E7/E6E7 RST). Resistant and double fluorescent clones (i.e. CFP/DsRed for H1 to H6, and GFP/DsRed for IB105/106) were amplified (Supplementary Fig. 1, A and B) for further genetic and cellular analyses. All hybrids were shown to result from fusion events, and harbored a highly rearranged genome, together with new migration capacities and aggressiveness in vivo [9].

### Gene expression profiling

Gene expression analysis was carried out using Agilent Whole human 44 K Genome Oligo Array (Agilent Technologies) according to the manufacturer’s protocol. All microarrays were simultaneously normalized using the Quantile algorithm. T-tests were performed using Genespring (Agilent Technologies) and *P*-values were adjusted using the Benjamini-Hochberg procedure. The *P*-value and fold change cut-off for gene selection were 0.001 and 2, respectively. Gene ontology (GO) analysis was performed to establish statistical enrichment in GO terms using Genespring (Agilent Technologies). Heatmap and boxplot were performed using with R (v3.6.2). The datasets generated and analyzed are available at the GEO, https://www.ncbi.nlm.nih.gov/geo/query/acc.cgi?acc=GSE171471.

### Cell proliferation assay

Cells were seeded in 96-well plates at a concentration of 2000 cells/well (6 wells/per cell line) and treated with AICAR (700 μM) or vehicle (H_2_O) 24 h later. RPMI galactose medium was prepared as indicated in [27]. The number of cells was evaluated by flow cytometry (FACS Calibur, BD Biosciences) at day 6. Data were analyzed using FlowJo (Tree Star, Celeza GmbH) and GraphPad (La Jolla, CA) software programs. For each cell line, the average number of cells present in AICAR-treated wells at day 6 was calculated and expressed as a percentage of growth compared to the corresponding control wells (average number of cells present in the wells of the untreated control = 100% of growth).

### Migration assay

Migration assays were performed using the IncucyteS3 Live-cell analysis system (Essen BioScience, Hertfordshire, UK). Briefly, cells were seeded in 96-well ImageLock plates (3 × 10^4^ cells for IB105 DsRed, IB106 GFP and 2 × 10^4^ for IB105/106 hybrids) and treated 24 h later with vehicle or AICAR (700 μM) for 24 h (12 wells/condition for each cell line). After removal of AICAR, a strip of cells was removed from each well using the WoundMaker device following the manufacturers’ instructions. The plate was then placed in the IncucyteS3 machine (37 °C and 5% CO_2_) for 24 h. Images were taken every 4 h for 24 h with a 10x objective. Cell migration analysis was done using the Incucyte ZOOM software (Essen BioScience, Hertfordshire, UK) and expressed as percentage relative wound density (RWD (%)), a metric taking into account the spatial cell density in the wound area relative to the spatial cell density outside of the wound area at every time point. RWD values are self-normalizing for changes in cell density which may occur outside the wound due to cell proliferation and/or pharmacological effects. Data plotting and statistical analysis was performed with GraphPad (La Jolla, CA) software.

### Invasion assay

Invasion was monitored using Matrigel-coated cell culture inserts containing 8.0 μm pore transparent positron emission tomography membranes (Fisher Scientific). AICAR (700 μM) or vehicle pre-treatments were done into 6-well plates seeded with 10^5^ of parental or hybrid cells. After 24 h of contact, control and AICAR-treated cells were collected and added to 24-well Boyden chamber plates (25,000 cells/well) in presence of RPMI-1640 (Gibco, Invitrogen) plus 5% FBS (upper chamber). The lower chamber was filled with RPMI-1640 plus 10% FBS to create a gradient supporting cell invasion. After 18 h of incubation, cells located at the top side of the membrane were removed using cotton-tipped swab, while invasive cells located at the other end were fixed with cold ethanol absolute for 15 min and stained with Hoechst 33,342 (1/5000) for 10 min at room temperature. For each insert the entire bottom membrane was acquired using an Axio Vert.A1(ZEISS) microscope. Quantification was done using the Image J cell counter plugin. Data were normalized according to the number of cells at time zero and 18 h evaluated in parallel (in mirror 24-well plates) by flow cytometry and plotted using GraphPad (La Jolla, CA) software.

### Glucose quantification

5 × 10^3^ cells of E6E7, RST and H1-H6 hybrids were seeded in 24-well plates. An aliquot of cell culture supernatant was removed every 24 h from day 0 to day 4 and kept for evaluation of glucose. The number of cells in the corresponding wells was determined each day by flow cytometry. Doubling times were then calculated using exponential curves in Excell software. Glucose was measured enzymatically in protein free extracts by spectrophotometric determination according to Bergmeyer [48]. Data were plotted to generate glucose consumption curves. Linear equations were then defined for each cell line using Excell software and used to determine the quantity of glucose used produced by each line per doubling time.

### Respiratory rate measurement

Oxygen consumption was measured at 37 °C using a Clark-type electrode (Oxygraph, Oroboros Instruments). The cells were pelleted at 1200 rpm for 5 min, resuspended in 200 μl of RPMI 1640 and added to the measurement chambers pre-filled with 2.1 ml of RPMI-1640/Hepes (10 mM) medium. Basal respiration was recorded, after stabilization of the electrode, in presence of 5 mM pyruvate for 10–15 min. Sequential additions of oligomycin (0,25 μg/ml) or dinitrophenol (DNP, 50-100 μM) were then added to monitor minimal (without oxidative phosphorylation) and maximal respiration rate, respectively. Citrate synthase activity was assayed according to the method described by Srere (Faloona and Srere, 1969) for normalization of the data. The reaction mixture contained 50 mM Tris buffer (pH 7.5), 0.1 mM EDTA, 0.1 mM acetyl-CoA, 0.2 mM 5,5-dithiobis (2-nitrobenzoic acid) (DTNB), 0.05% triton X-100, and the enzyme extract. The reaction was initiated with 0.5 mM oxaloacetate and monitored at 412 nm for 10–15 min at 25 °C. The enzyme activity was calculated using an extinction coefficient of 13,600 M^− 1^ cm^− 1^ at 412 nm.

### Western blot

Protein extracts were prepared as described in [49]. AMPKα protein expression level was evaluated using a primary antibody directed against AMPKα (Cell signaling technology #2532). This antibody recognizes alpha 1 and alpha 2 isoforms of the catalytic subunit, but not the regulatory beta subunit. 20 *μ* g of proteins were loaded on the gel and separated by SDS-page. After the transfer onto a PVDF membrane, the membrane was blocked in PBS-Tween 0.1% BSA 5% buffer and then incubated with the primary antibody (1:1000) overnight at 4 °C. After several washes, the membrane was then incubated with a horseradish-peroxidase-linked anti-rabbit antibody (Cell signaling technology #12–2018) for 1 h at room temperature. Signal was detected using PXi (Syngene, Cambridge, UK) after incubation with chemiluminescent substrate (ECL Immobilon Western, WBKLS0100, Merck, Darmstadt, Germany). β-actin (Sigma #A5316, 1:10000, 1 h at room temperature) with an anti-mouse secondary antibody (Cell signaling technology #08–2018; 1:10000, 1 h at room temperature) was used as a loading control for quantification.

## Supplementary Information

**Additional file 1: Supplementary Figure 1.** E6E7/RST fusion model. (A) Schematic representation of H1 to H6 hybrid generation. IMR90 E6E7-CFP and IMR90 E6E7-RST-DsRed parental cell lines were co-cultured for 72 h prior to antibiotic selection (blasticidin/puromycin) to obtain pure hybrid cell lines. (B) Schematic representation of IB105/IB106 hybrid generation. IB105 DsRed and IB106 GFP were co-cultured for 72 h and pure IB105/106 hybrid cells were obtained after 3 successive rounds of cell sorting and amplification. **Supplementary Figure 2.** Respiratory rate. (A) Respiratory rate of E6E7, RST and H1-H6 hybrids under oligomycin (upper left) and 2,4 dinitrophenol (DNP) (upper right). Values were normalized to the number of cells and expressed in nmol of O2 consumed per minute for a million cells. Experiments have been repeated 2 to 7 times, according to the sample and treatment. Lower left: Values of the ratio VO_2_ pyr / VO_2_ oligo for each parental and hybrid cell line. Lower right: Respiratory state value for each parental and hybrid cell line. Statistical analyses were done using one-way ANOVA test followed by Holm-Sidak multiple comparison test (*p < 0.05, **p < 0.01, ***p < 0.001; error bars, SD). (B) Citrate synthase normalized respiratory rates as presented in (a). **Supplementary Figure 3.** Example of determination of the glucose consumption production rates. (A) Glucose consumption curve of E6E7, RST and H1-H6 hybrids from day 0 to day 4. The graph corresponds to the results obtained for one experiment out of two (values in the graph obtained from duplicates). For each sample, glucose consumption rate was established as following a linear equation (*n* = 2 in duplicates). (B) Growth curves of E6E7, RST and H1-H6 hybrids determined by cell counting using flow cytometry. Samples used here correspond to the ones used in (A). Doubling times were established using exponential curve equations in Excell software. **Supplementary Figure 4.** (A) Growth of E6E7, RST and H1-H6 hybrids cells in glucose vs galactose supplemented RPMI evaluated by flow cytometry at day 5 post-treatment. Data are expressed as a percentage of growth compared to the control (growth in RPMI+GLU = 100%). Statistical analyses were performed using an unpaired t-test (*p < 0,05; **p < 0,01, ****p < 0,0001; error bars, SD, *n* = 3 in triplicates). (B) Growth of E6E7, RST and H1-H6 hybrids cells in RPMI supplemented with 2, 0.4 or 0 mM of glutamine evaluated by flow cytometry at day 3 post-treatment. Data are expressed as a percentage of growth compared to the control (growth in RPMI 2 mM of glutamine = 100%). Statistical analyses were performed using an ordinary one way ANOVA test followed by a muticomparison Dunnett (compared to glut 2 mM sample: *p < 0,05; **p < 0,01; error bars, SD, *n* = 2 in triplicates). **Supplementary Figure 5.** AMPKα1/ α2 protein expression in parental and hybrid cell lines (A) Protein level expression of AMPKα1/ α2 at protein level by western blot. Expression at 62 kDa pointed by black arrow. (B) Quantification of protein using monoclonal antibody β-actin and using RST to normalize. **Supplementary Figure 6.** IB105 and IB105/106 migration capacities are reduced in presence of AICAR. (A) Images of IB105, IB-106 and IB105/106 pre-treated or not with AICAR after a wound healing assay performed with the IncucyteS3. Black box represents the size of the would at time zero, the red dashed line represents the size of the wound at 8 h and the red dotted line represents the size of the wound at 24 h. The experiment was performed twice with twelve replicates. (B) Images obtained in (A) were processed using the wound mask from the IncucyteS3 software. IncucyteS3 software calculates the area cover by cells (yellow) and the area of the wound (grey) at each time point.

**Additional file 2.**

**Additional file 3.**

## Data Availability

The datasets generated and analyzed during the current study are available in the GEO repository, https://www.ncbi.nlm.nih.gov/geo/query/acc.cgi?acc=GSE171471 (token: sfetyawohlirluf).

## Abbreviations

UPS: Undifferentiated pleomorphic sarcoma
AICAR: 5-aminoimidazole-4-carbox-amide-1-β-D-ribofuranoside
AMP: 5′-adenosine monophosphate
AMPK: (AMP)-activated protein kinase
