# Supplementary material for: Cell fusion enhances energy metabolism of mesenchymal tumor hybrid cells to sustain their proliferation and invasion
Source: BMC Cancer. 2021 Jul 28;21:863. doi: 10.1186/s12885-021-08561-6 (PMC8317390; doi:10.1186/s12885-021-08561-6)
Supplement: Supplementary file 1 — Additional file 1: Supplementary Figure 1. E6E7/RST fusion model. (A) Schematic representation of H1 to H6 hybrid generation. IMR90 E6E7-CFP and IMR90 E6E7-RST-DsRed parental cell lines were co-cultured for 72 h prior to antibiotic selection (blasticidin/puromycin) to obtain pure hybrid cell lines. (B) Schematic representation of IB105/IB106 hybrid generation. IB105 DsRed and IB106 GFP were co-cultured for 72 h and pure IB105/106 hybrid cells were obtained after 3 successive rounds of cell sorting and amplification. Supplementary Figure 2. Respiratory rate. (A) Respiratory rate of E6E7, RST and H1-H6 hybrids under oligomycin (upper left) and 2,4 dinitrophenol (DNP) (upper right). Values were normalized to the number of cells and expressed in nmol of O2 consumed per minute for a million cells. Experiments have been repeated 2 to 7 times, according to the sample and treatment. Lower left: Values of the ratio VO2 pyr / VO2 oligo for each parental and hybrid cell line. Lower right: Respiratory state value for each parental and hybrid cell line. Statistical analyses were done using one-way ANOVA test followed by Holm-Sidak multiple comparison test (*p < 0.05, **p < 0.01, ***p < 0.001; error bars, SD). (B) Citrate synthase normalized respiratory rates as presented in (a). Supplementary Figure 3. Example of determination of the glucose consumption production rates. (A) Glucose consumption curve of E6E7, RST and H1-H6 hybrids from day 0 to day 4. The graph corresponds to the results obtained for one experiment out of two (values in the graph obtained from duplicates). For each sample, glucose consumption rate was established as following a linear equation (n = 2 in duplicates). (B) Growth curves of E6E7, RST and H1-H6 hybrids determined by cell counting using flow cytometry. Samples used here correspond to the ones used in (A). Doubling times were established using exponential curve equations in Excell software. Supplementary Figure 4. (A) Growth of E6E7, RST and H1-H6 hybr [file 12885_2021_8561_MOESM1_ESM.docx]

**Supplementary Figure 1**

(B)

Unique UPS

IB 106 GFP

Two cell lines

IB 105 DsRed

Hybrid IB105/106

(A)


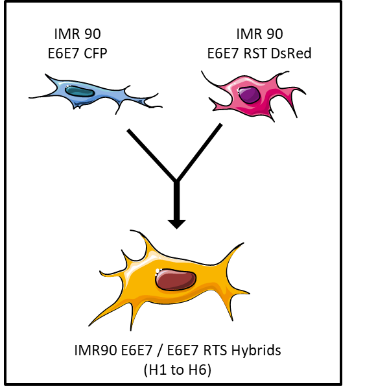


**Figure 1.** E6E7/RST fusion model. (A) Schematic representation of H1 to H6 hybrid generation. IMR90 E6E7-CFP and IMR90 E6E7-RST-DsRed parental cell lines were co-cultured for 72 hours prior to antibiotic selection (blasticidin/puromycin) to obtain pure hybrid cell lines. (B) Schematic representation of IB105/IB106 hybrid generation. IB105 DsRed and IB106 GFP were co-cultured for 72 hours and pure IB105/106 hybrid cells were obtained after 3 successive rounds of cell sorting and amplification.

**Supplementary Figure 2**

(A)

oligomycin

DNP

***

**

**

**

**

**

**

**

*

**

**

*

oligomycin

DNP

**

*

*

**

*

*

**

**

**

**

**

*

*

(B)

**Supplementary Figure 2. Respiratory rate.** (A) Respiratory rate of E6E7, RST and H1-H6 hybrids under oligomycin (upper left) and 2,4 dinitrophenol (DNP) (upper right). Values were normalized to the number of cells and expressed in nmol of O2 consumed per minute for a million cells. Experiments have been repeated 2 to 7 times, according to the sample and treatment. Lower left: Values of the ratio VO_2_ pyr / VO_2_ oligo for each parental and hybrid cell line. Lower right: Respiratory state value for each parental and hybrid cell line. Statistical analyses were done using one-way ANOVA test followed by Holm-Sidak multiple comparison test (*p<0.05, **p<0.01, ***p<0.001; error bars, SD). (B) Citrate synthase normalized respiratory rates as presented in (a)**.**

**Supplementary Figure 3**

(B)


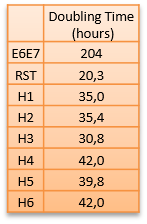


(A)

**Supplementary Figure 3.** Example of determination of the glucose consumption production rates. (A) Glucose consumption curve of E6E7, RST and H1-H6 hybrids from day 0 to day 4. The graph corresponds to the results obtained for one experiment out of two (values in the graph obtained from duplicates). For each sample, glucose consumption rate was established as following a linear equation (n=2 in duplicates). (B) Growth curves of E6E7, RST and H1-H6 hybrids determined by cell counting using flow cytometry. Samples used here correspond to the ones used in (A). Doubling times were established using exponential curve equations in Excell software.

**
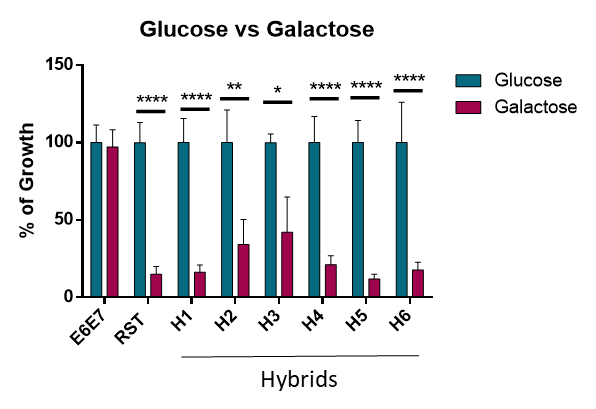
Supplementary Figure 4**

(A)

(B)

**
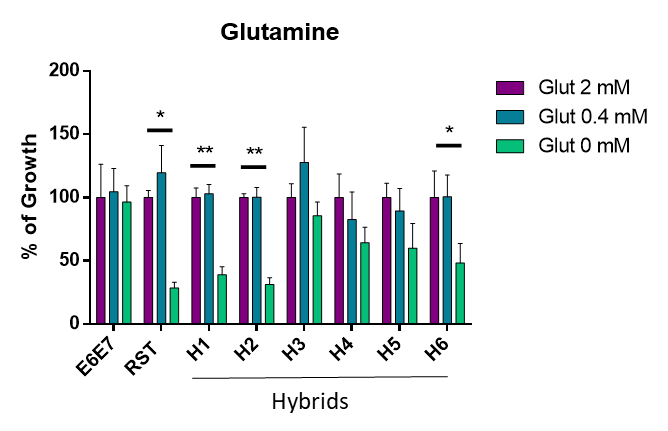
**

**Supplementary Figure 4.** (A) Growth of E6E7, RST and H1-H6 hybrids cells in glucose vs galactose supplemented RPMI evaluated by flow cytometry at day 5 post-treatment. Data are expressed as a percentage of growth compared to the control (growth in RPMI+GLU = 100%). Statistical analyses were performed using an unpaired t-test (*p<0,05; **p<0,01, ****p<0,0001; error bars, SD, n=3 in triplicates). (B) Growth of E6E7, RST and H1-H6 hybrids cells in RPMI supplemented with 2, 0.4 or 0 mM of glutamine evaluated by flow cytometry at day 3 post-treatment. Data are expressed as a percentage of growth compared to the control (growth in RPMI 2mM of glutamine = 100%). Statistical analyses were performed using an ordinary one way ANOVA test followed by a muticomparison Dunnett (compared to glut 2mM sample: *p<0,05; **p<0,01; error bars, SD, n=2 in triplicates).

**Supplementary Figure 5**

55 -


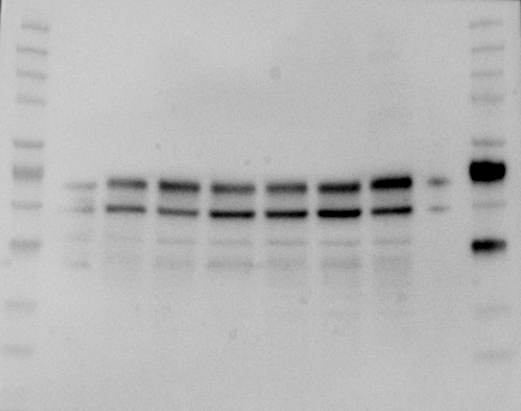


HeLa

E6E7

RST

H1

H2

H3

H4

kDa

70 -

40 -

AMPK


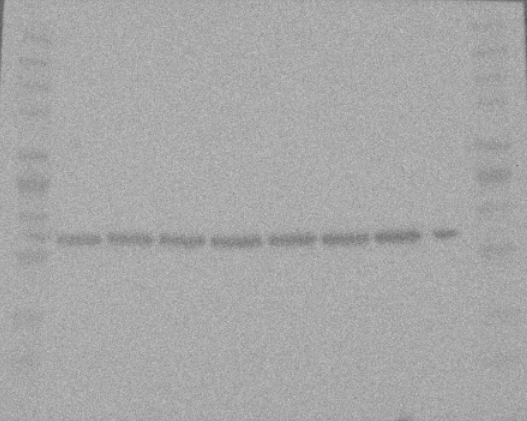


Actine

kDa

70 -

55 -

40 -

HeLa

E6E7

RST

H1

H2

H3

H4

(A)

**
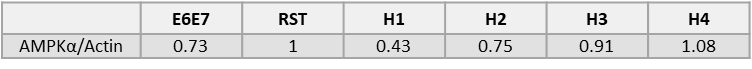
**

(C)

(B)

**Supplementary Figure 5.** AMPKα1/ α2 protein expression in parental and hybrid cell lines (A) Protein level expression of AMPKα1/ α2 at protein level by western blot. Expression at 62kDa pointed by black arrow. (B) Quantification of protein using monoclonal antibody β-actin and using RST to normalize. **Supplementary Figure 6**

(A)

(B)

IB105

non-treated

IB105/106

non-treated

IB106

non-treated

T0

T8

T24

IB105

AICAR

IB106

AICAR

IB105/106

AICAR


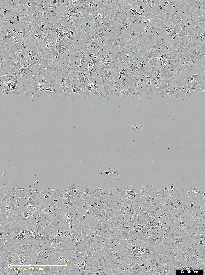

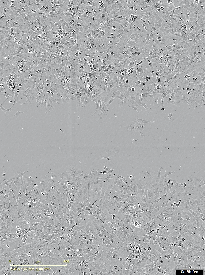

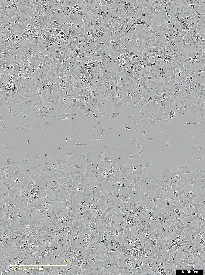

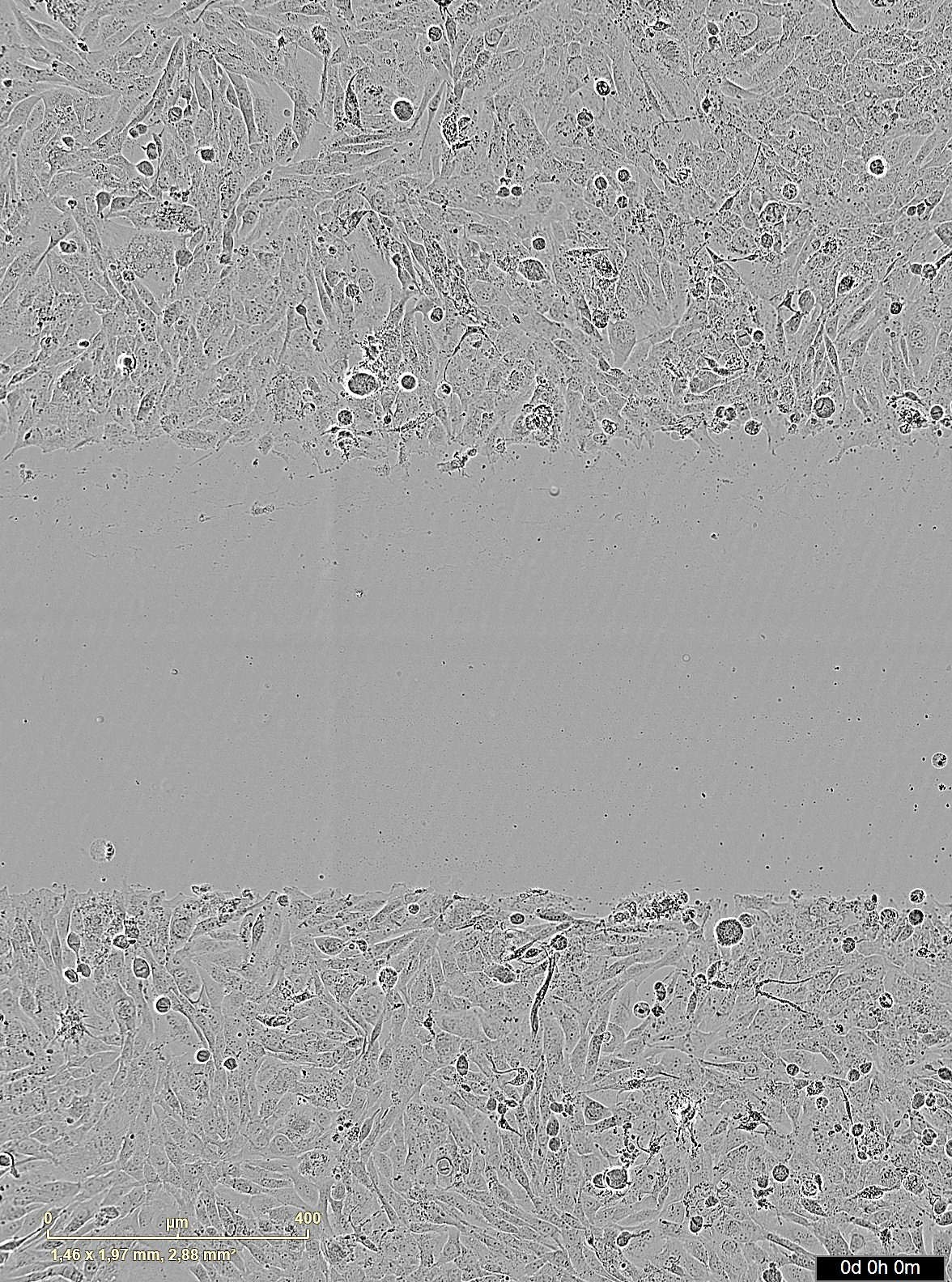

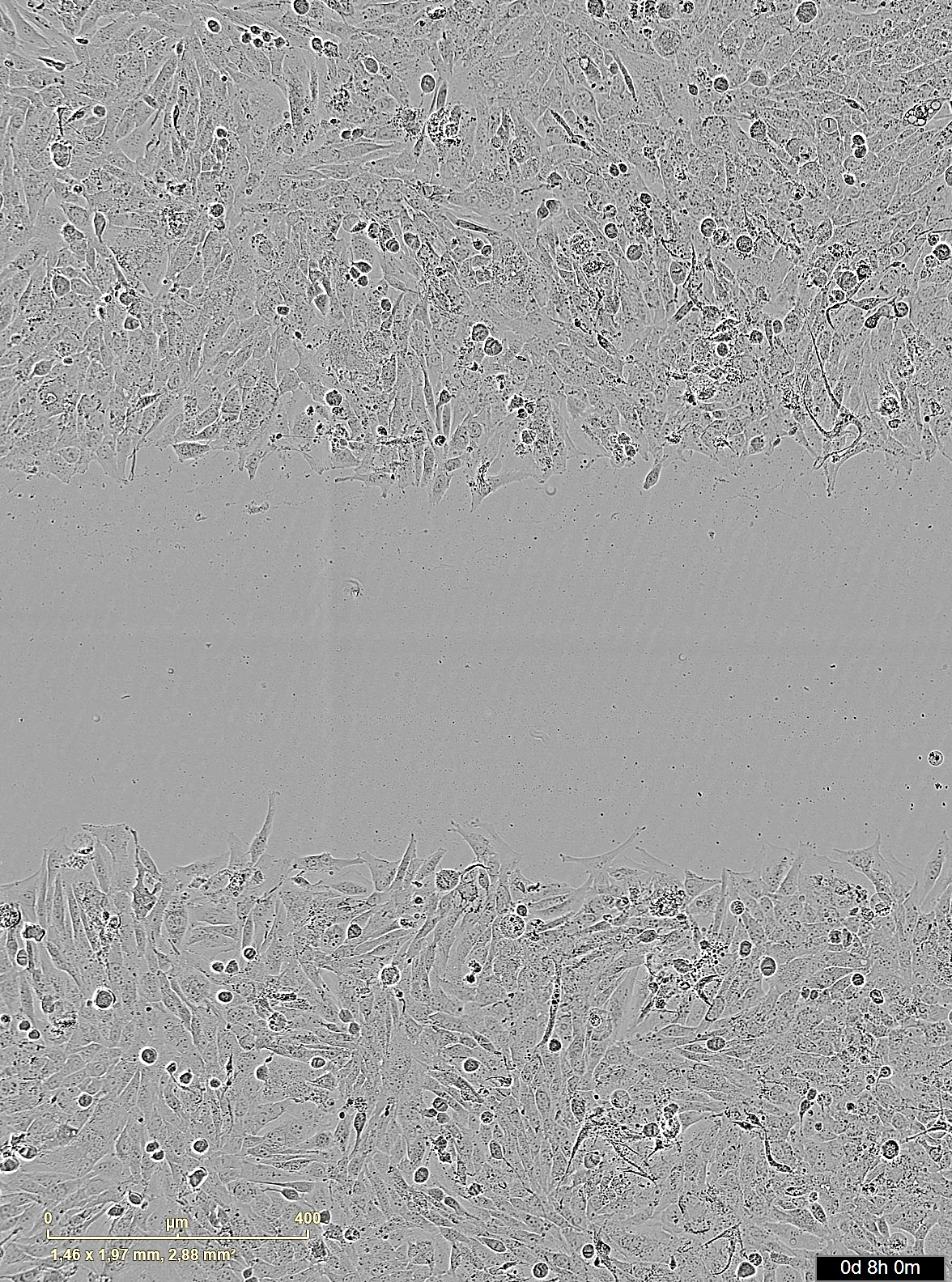

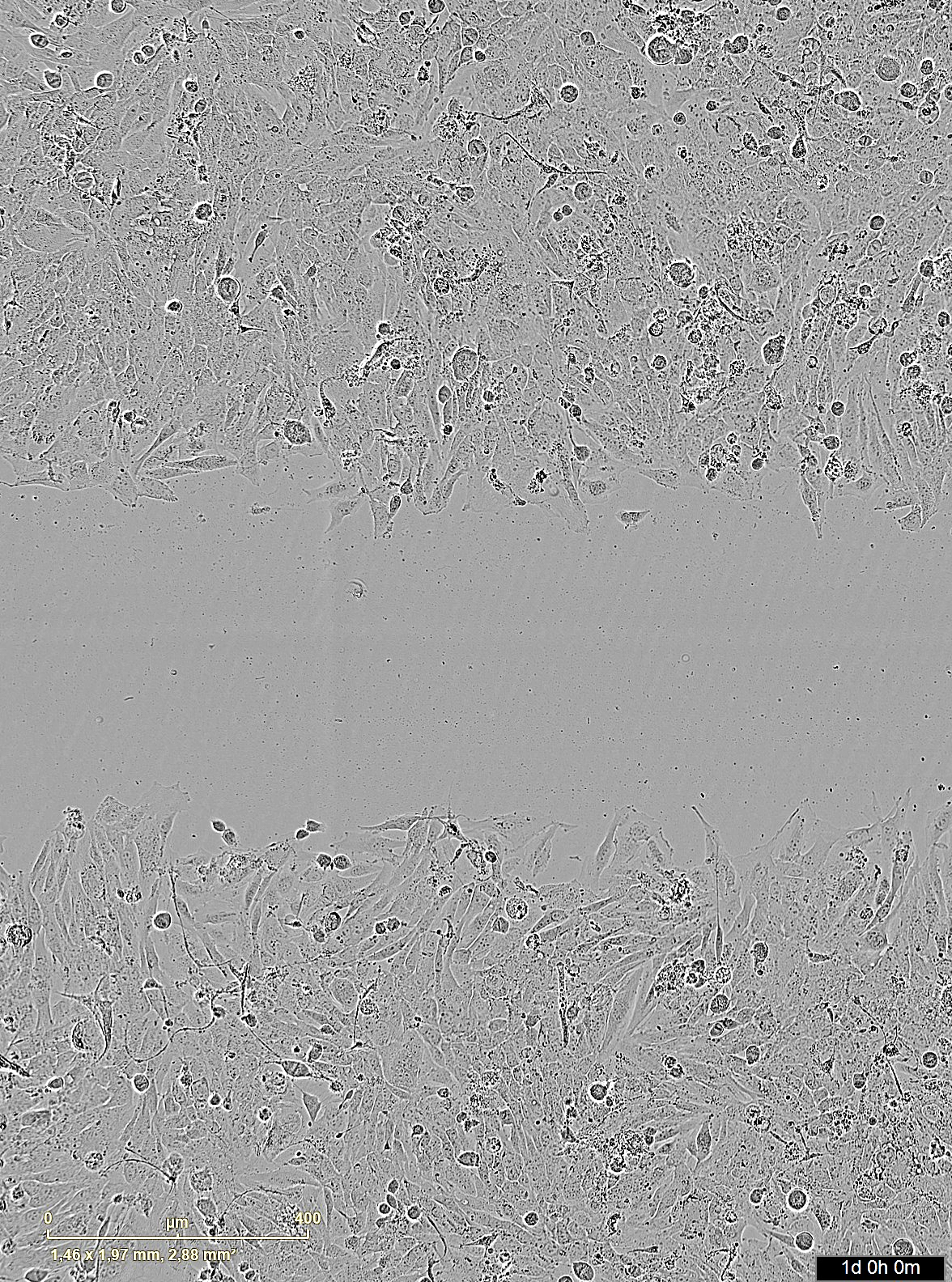

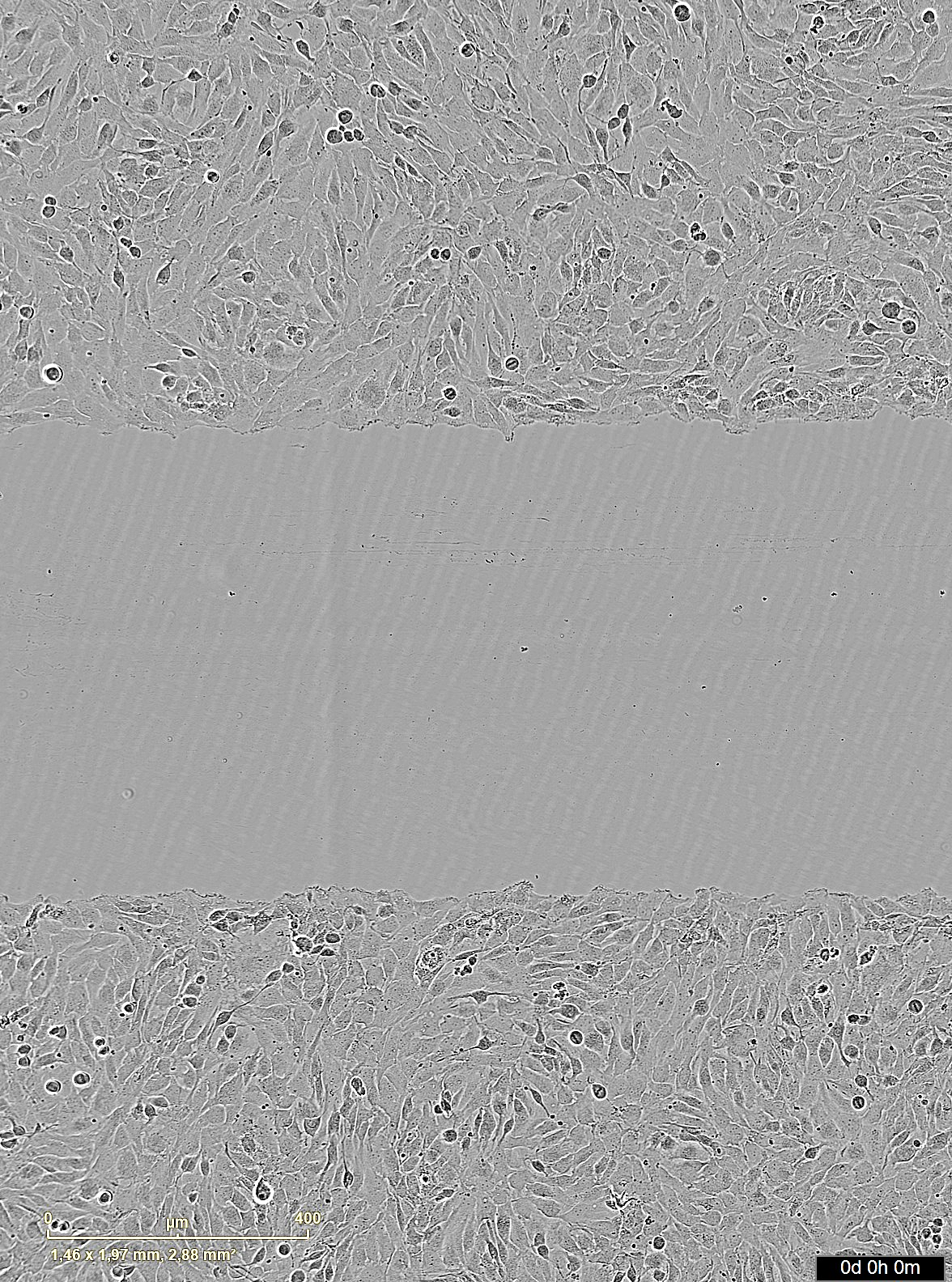

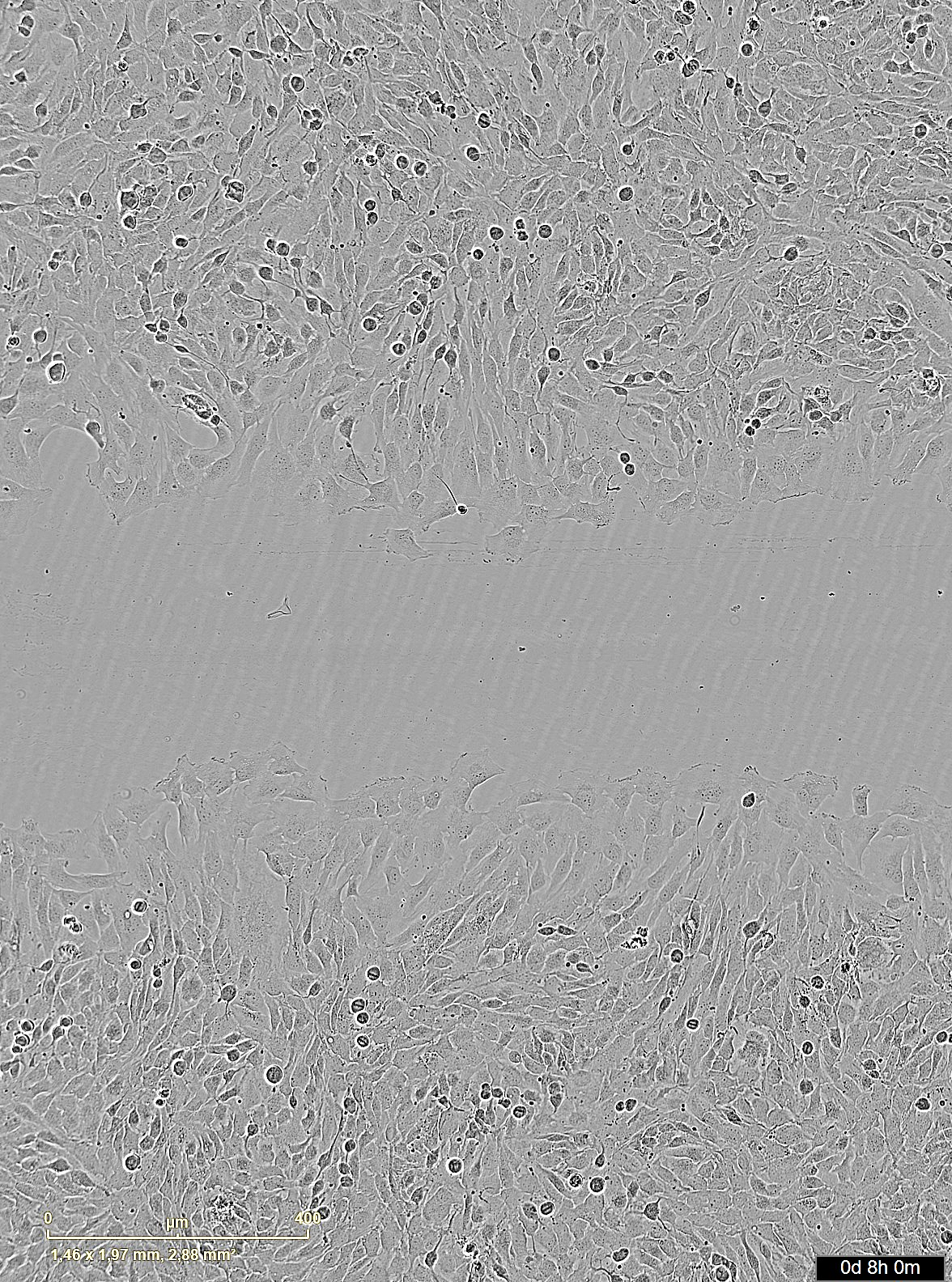

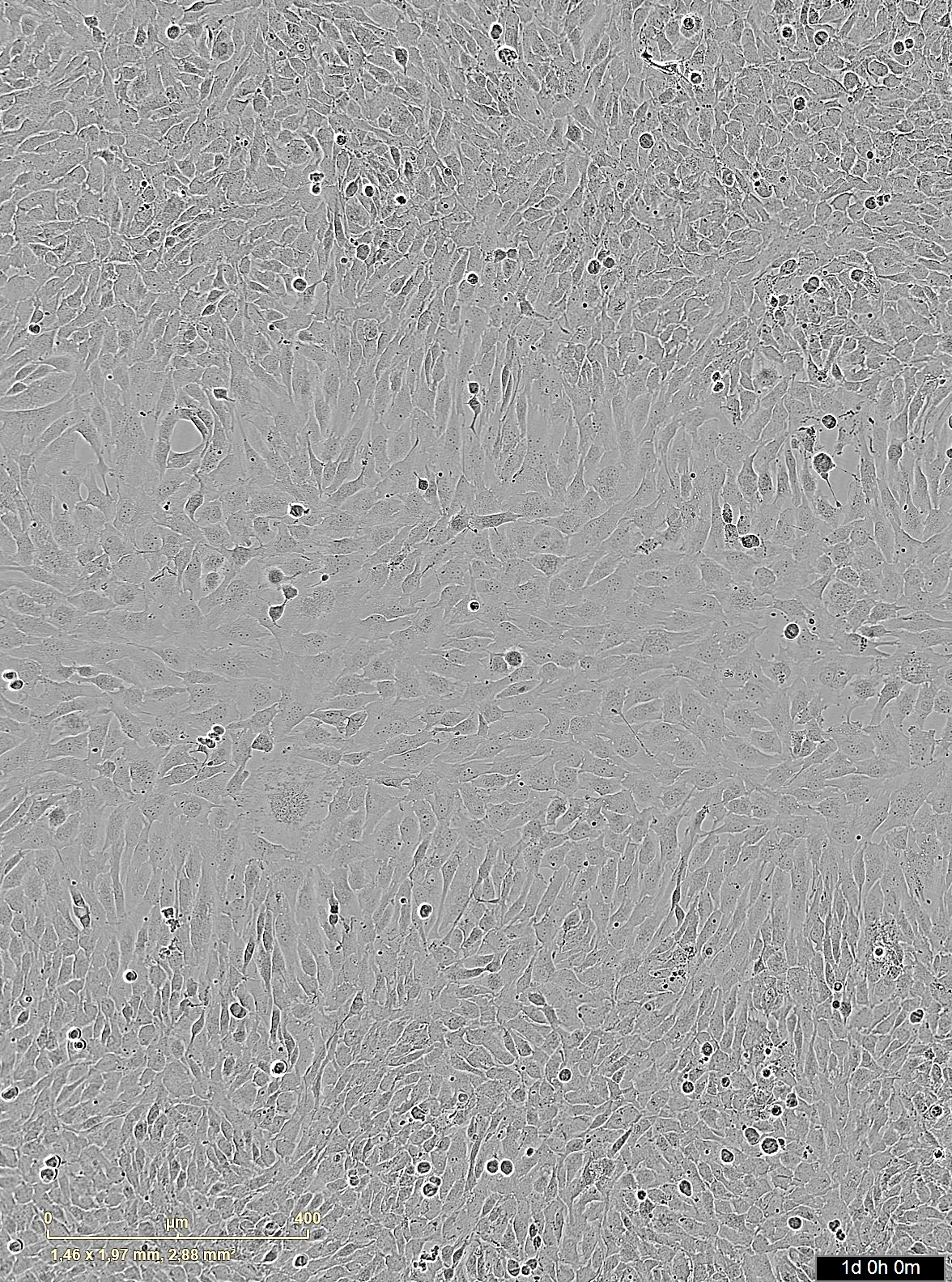

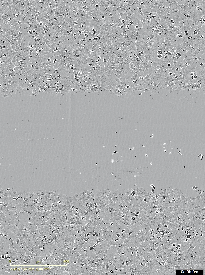

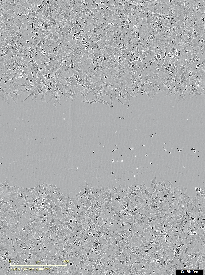

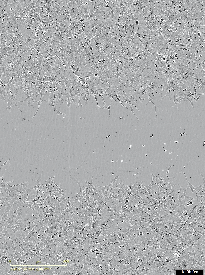

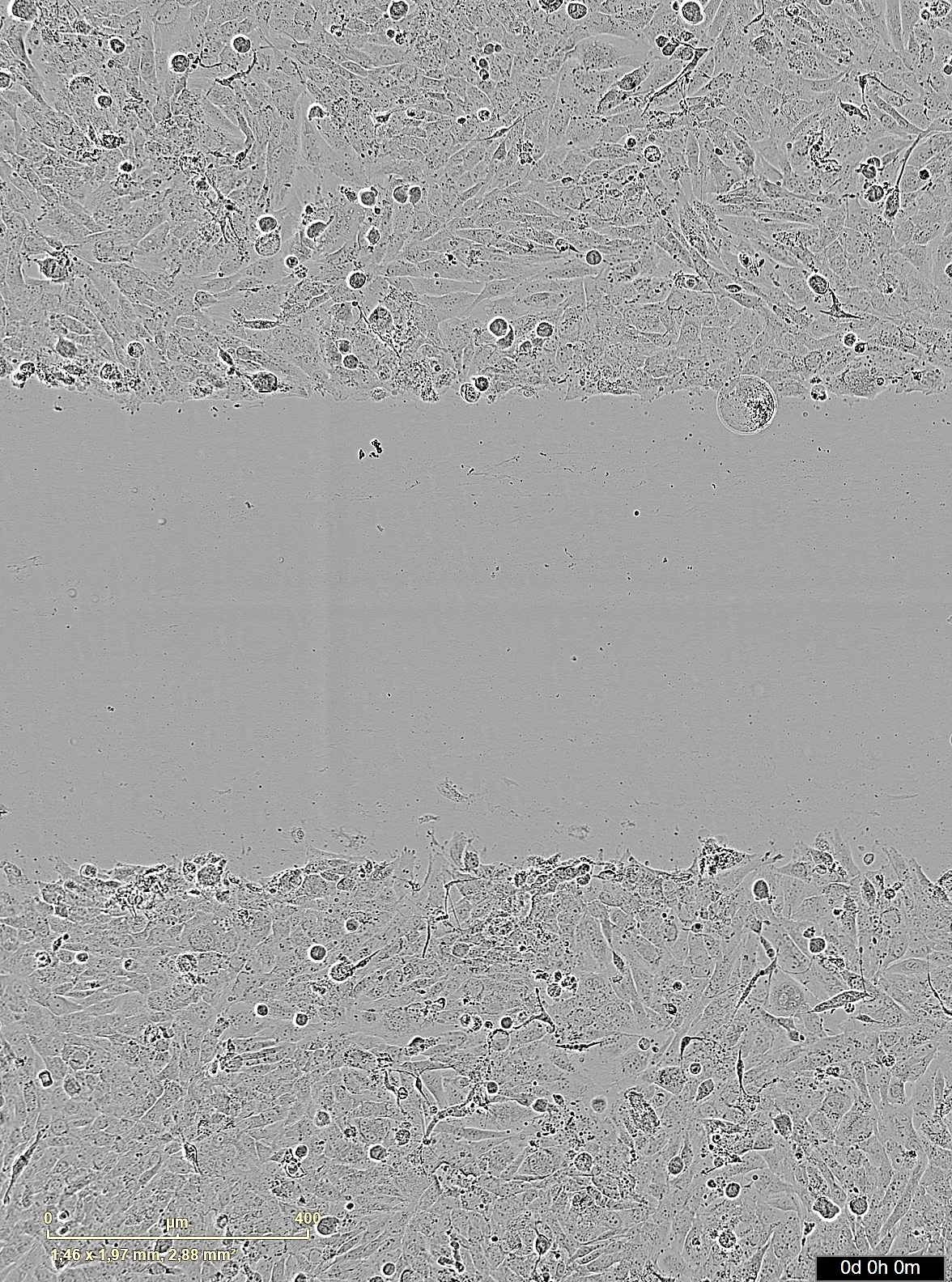

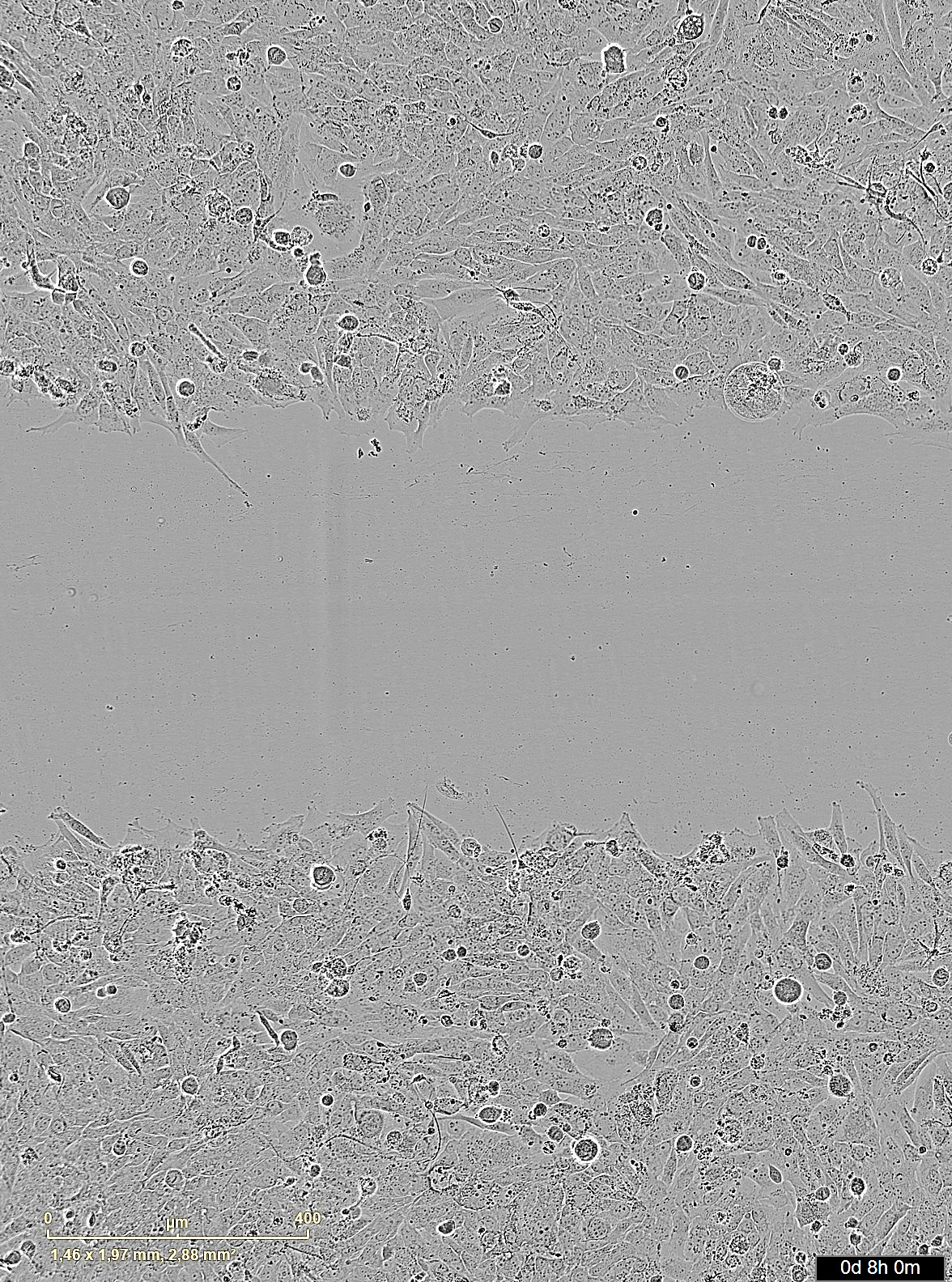

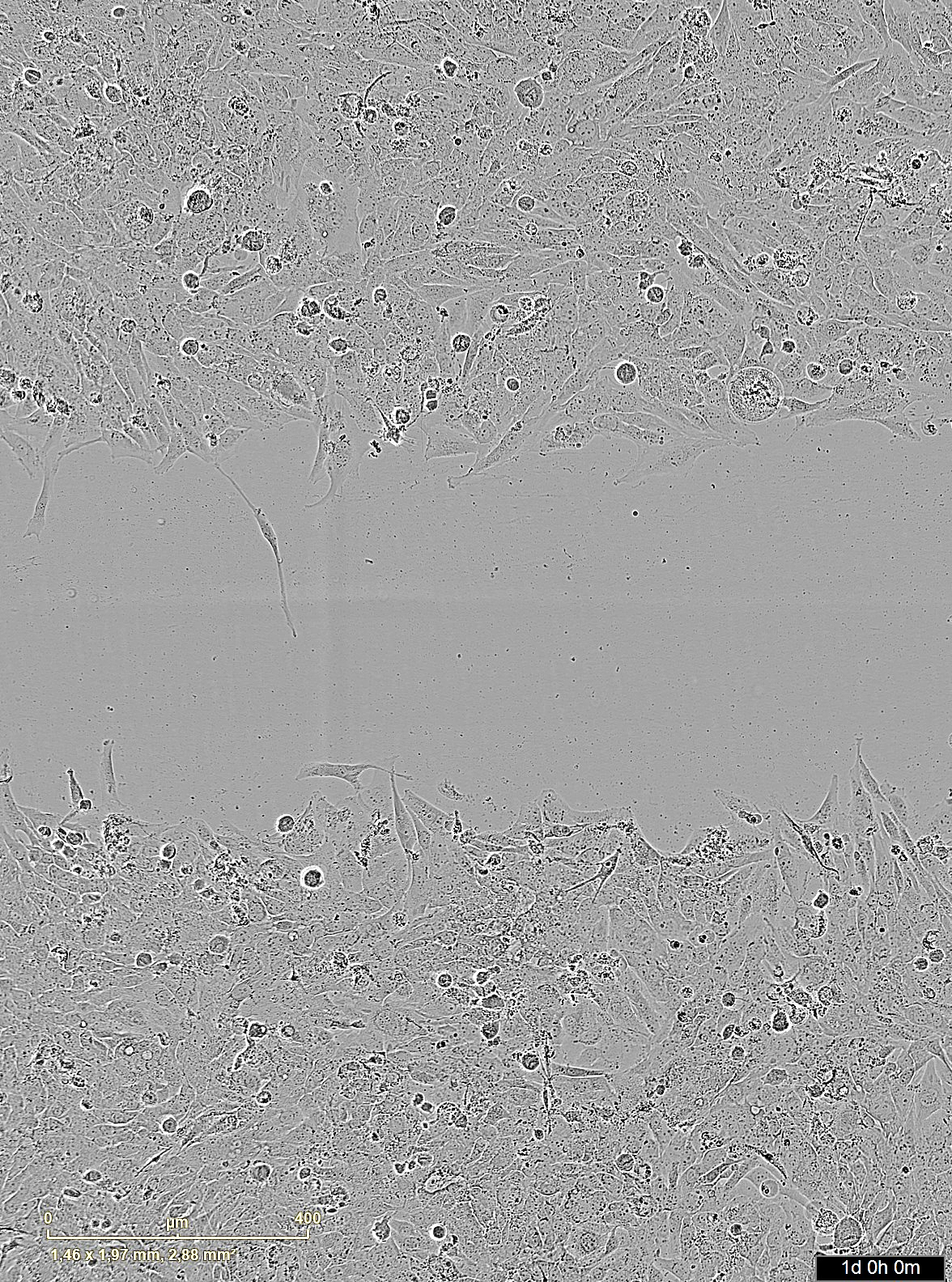

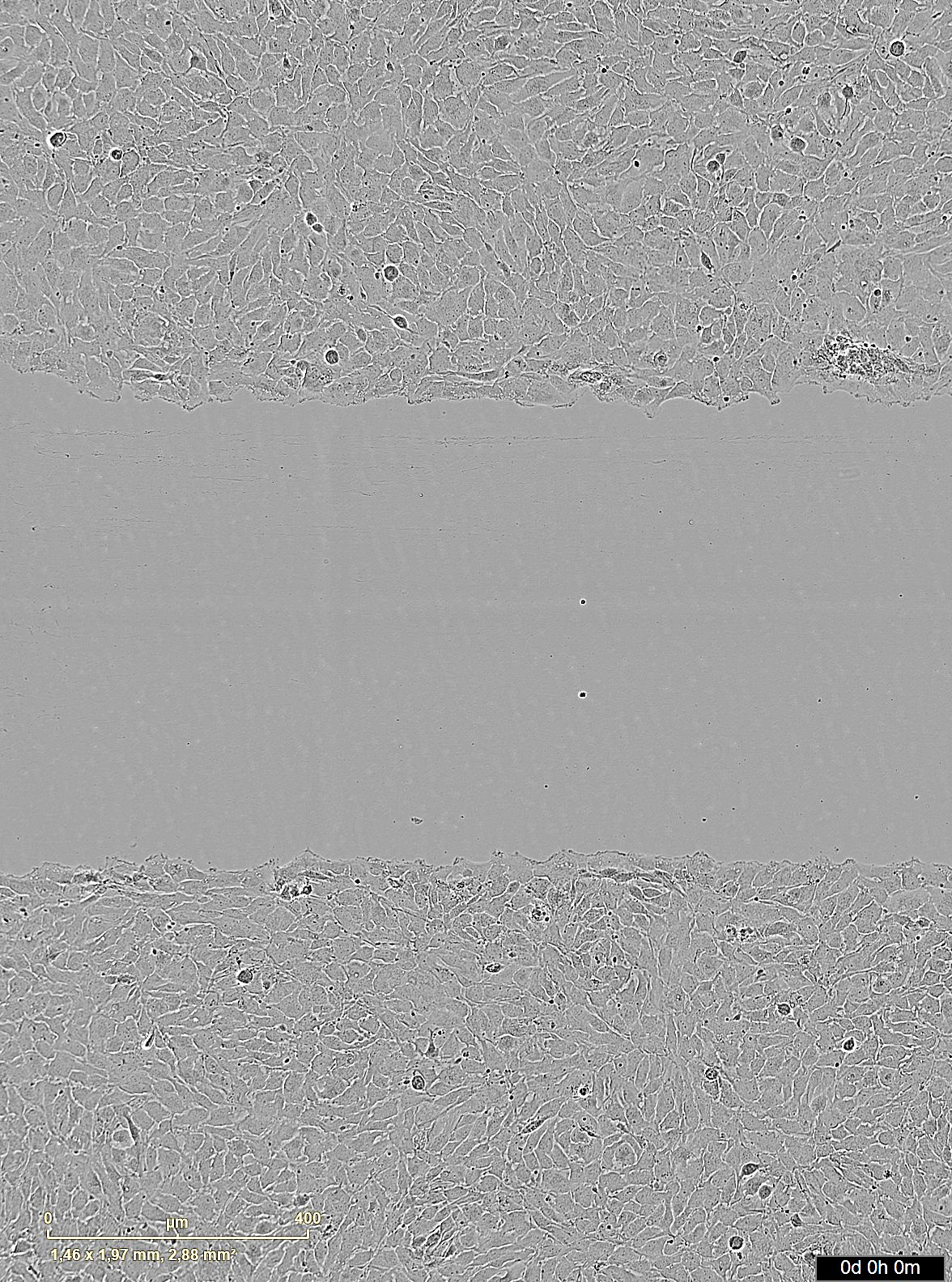

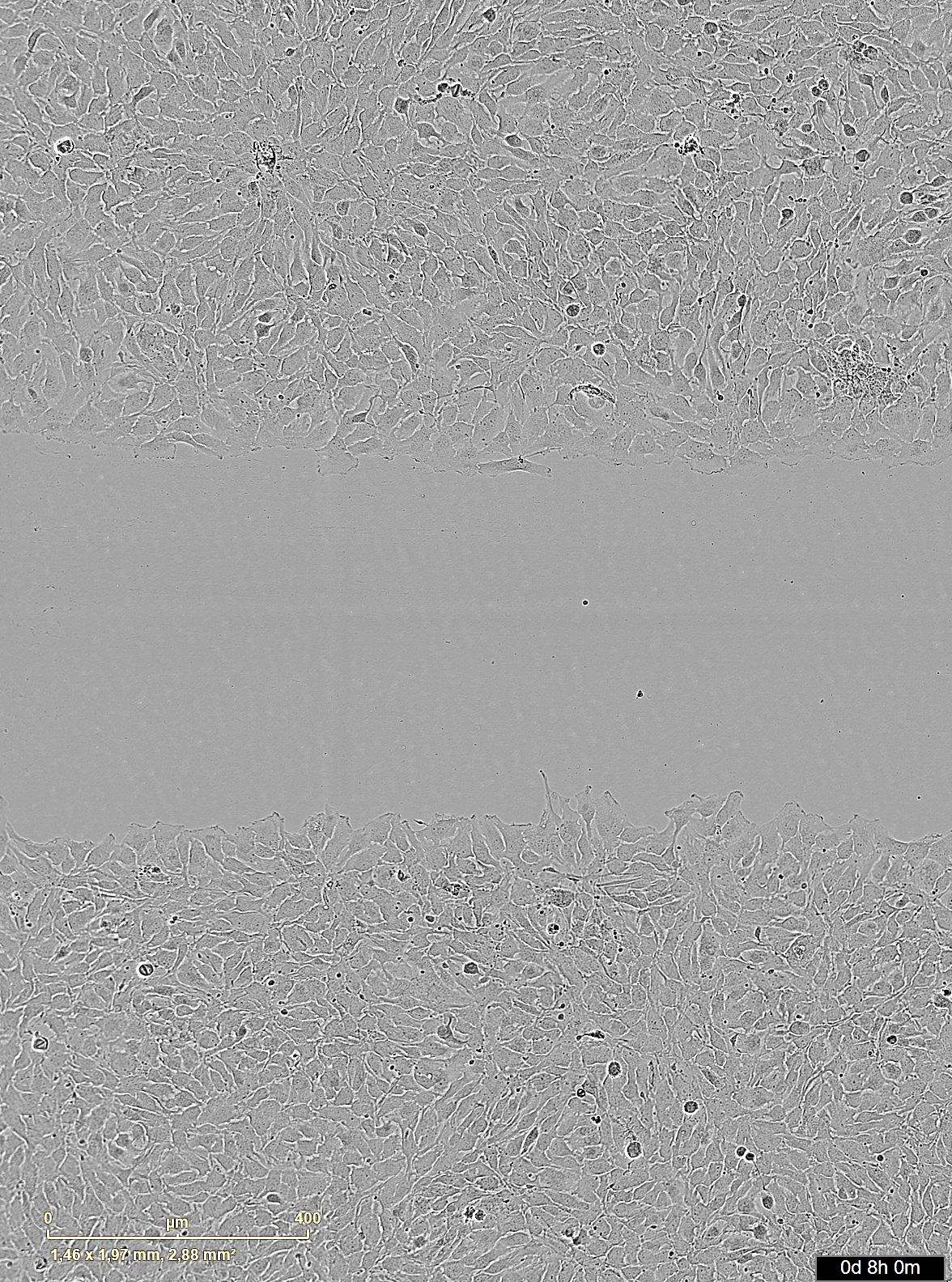

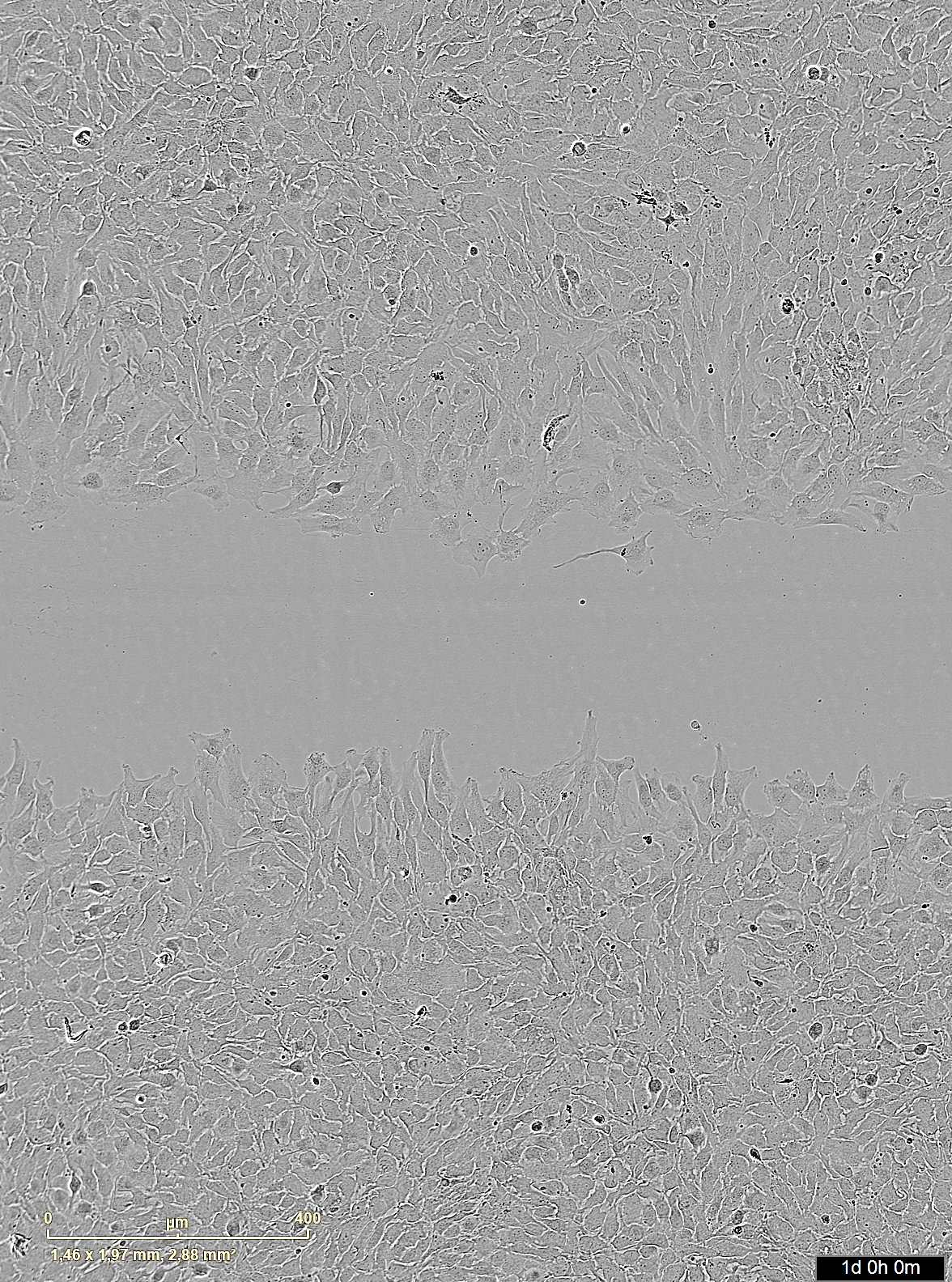


Parental cells

Hybrid

IB105

non-treated

IB105/106

non-treated

IB106

non-treated

T0

T8

T24

IB105

AICAR

IB106

AICAR

IB105/106

AICAR


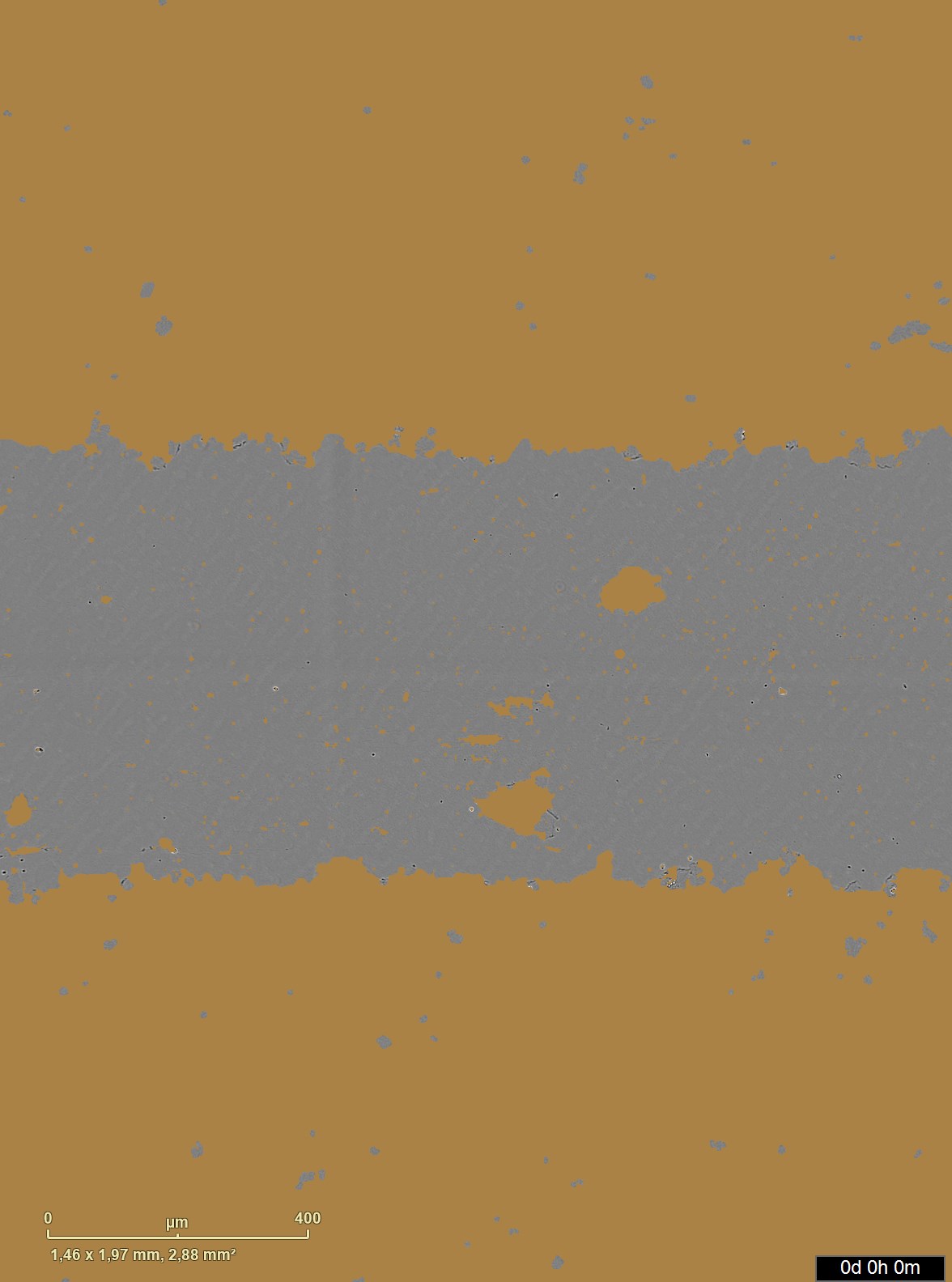

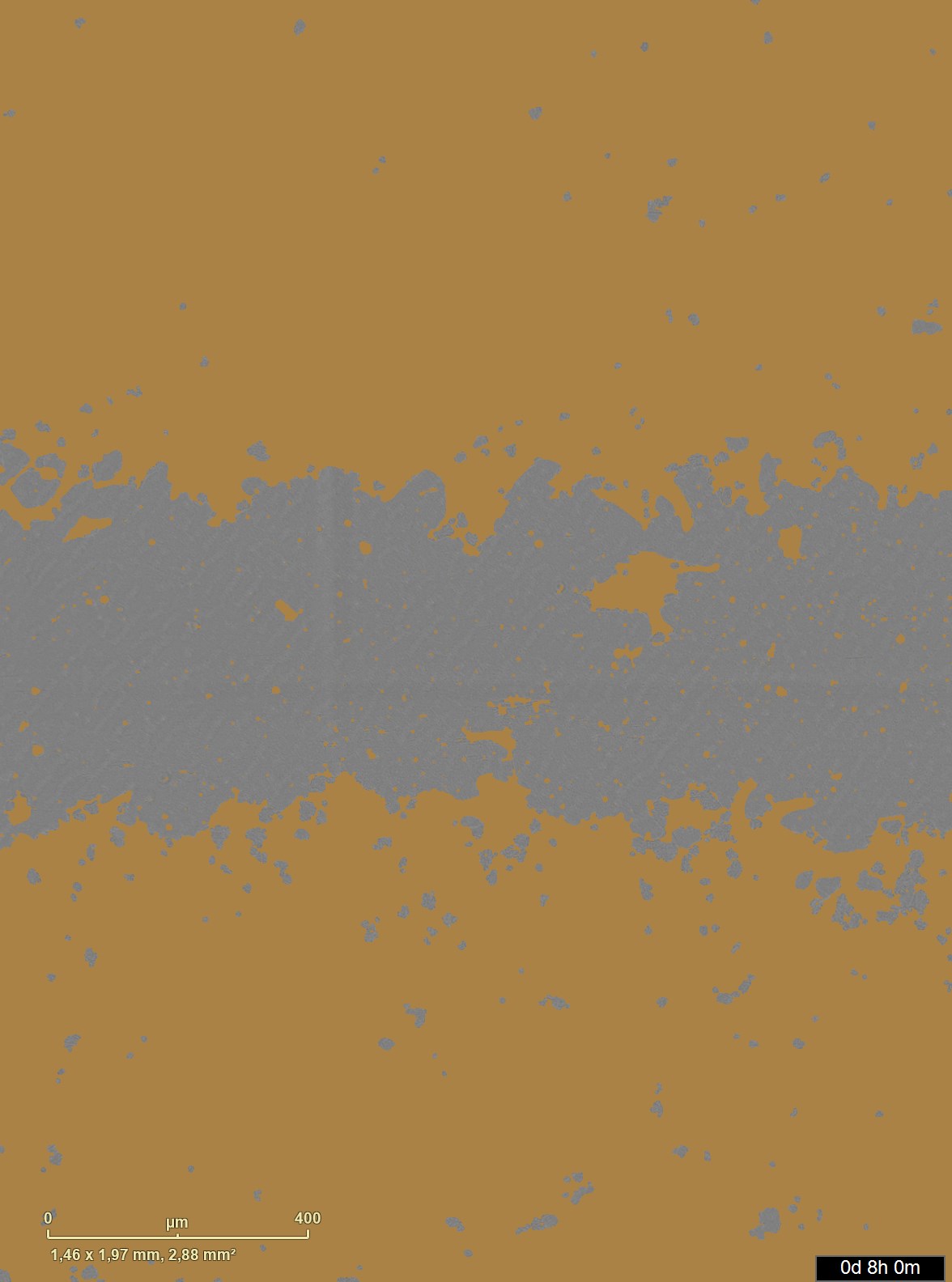

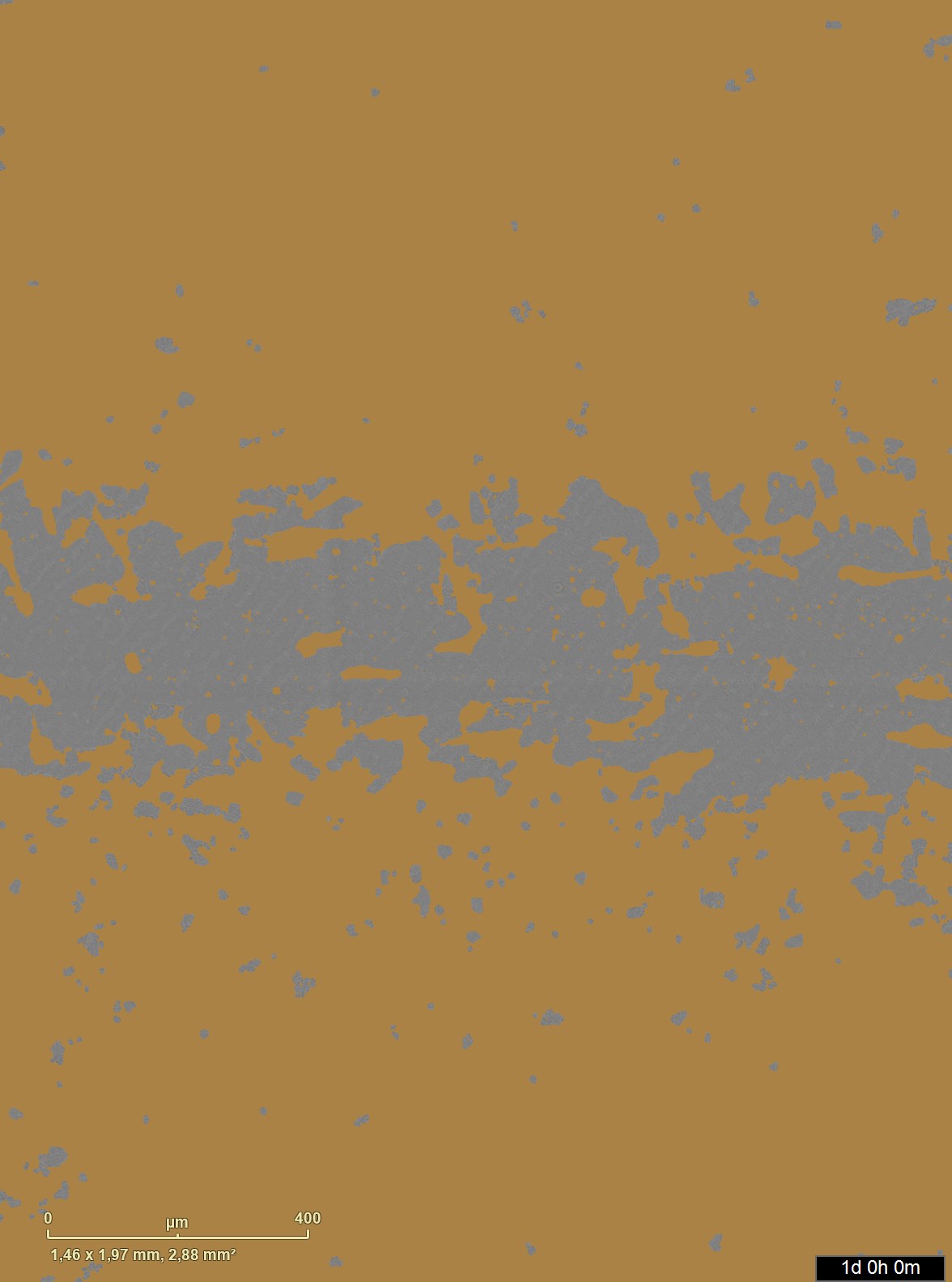

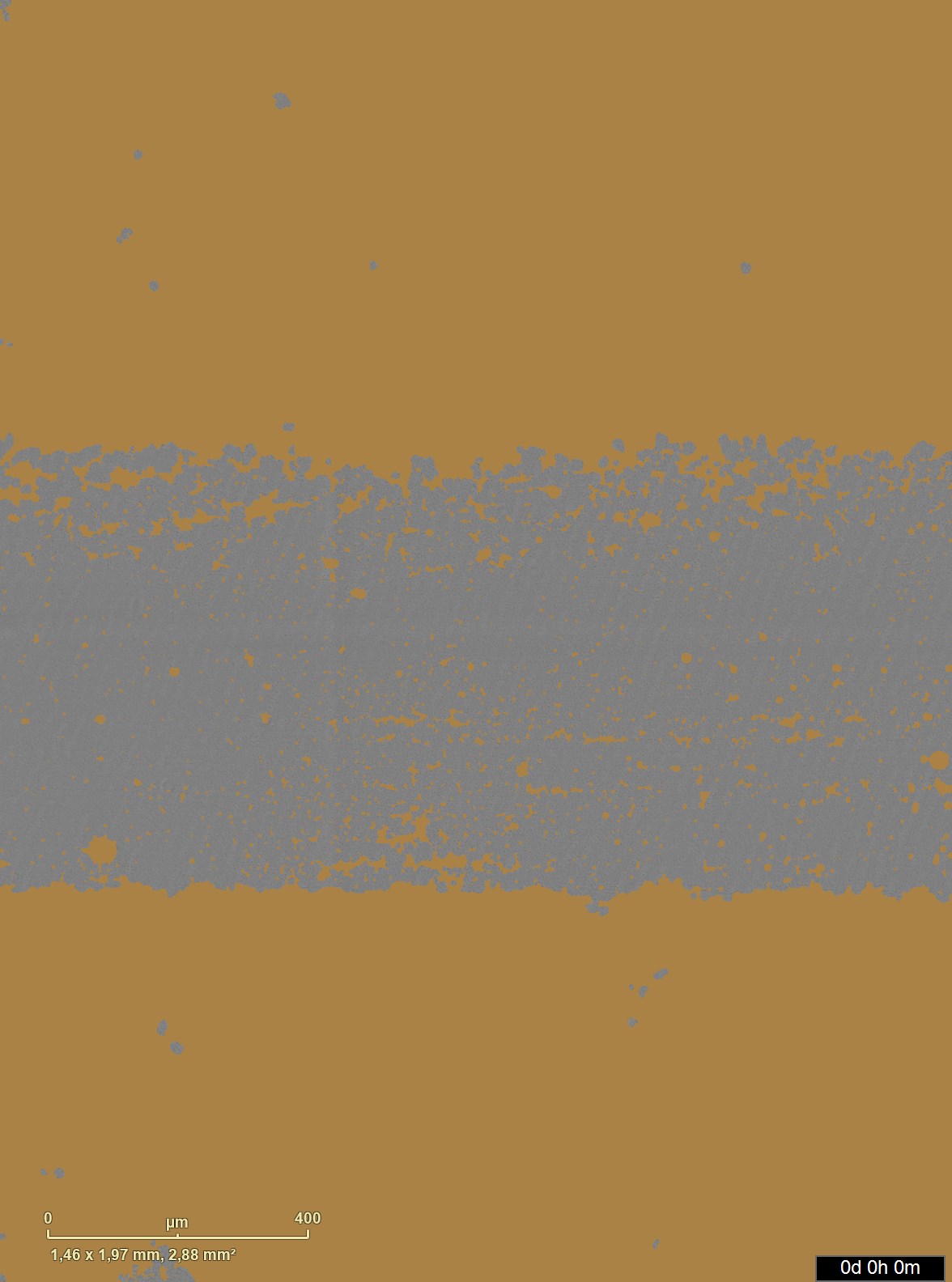

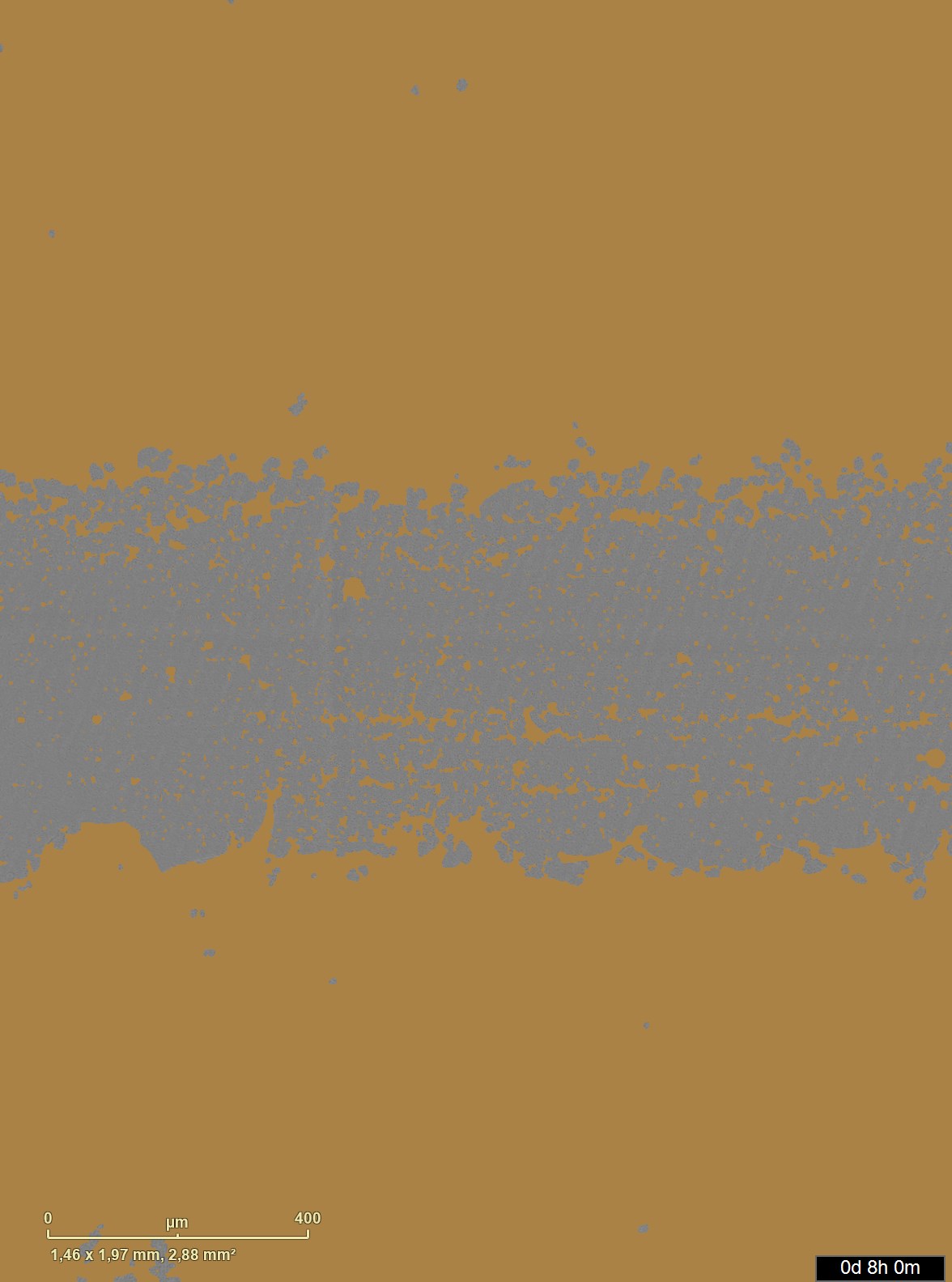

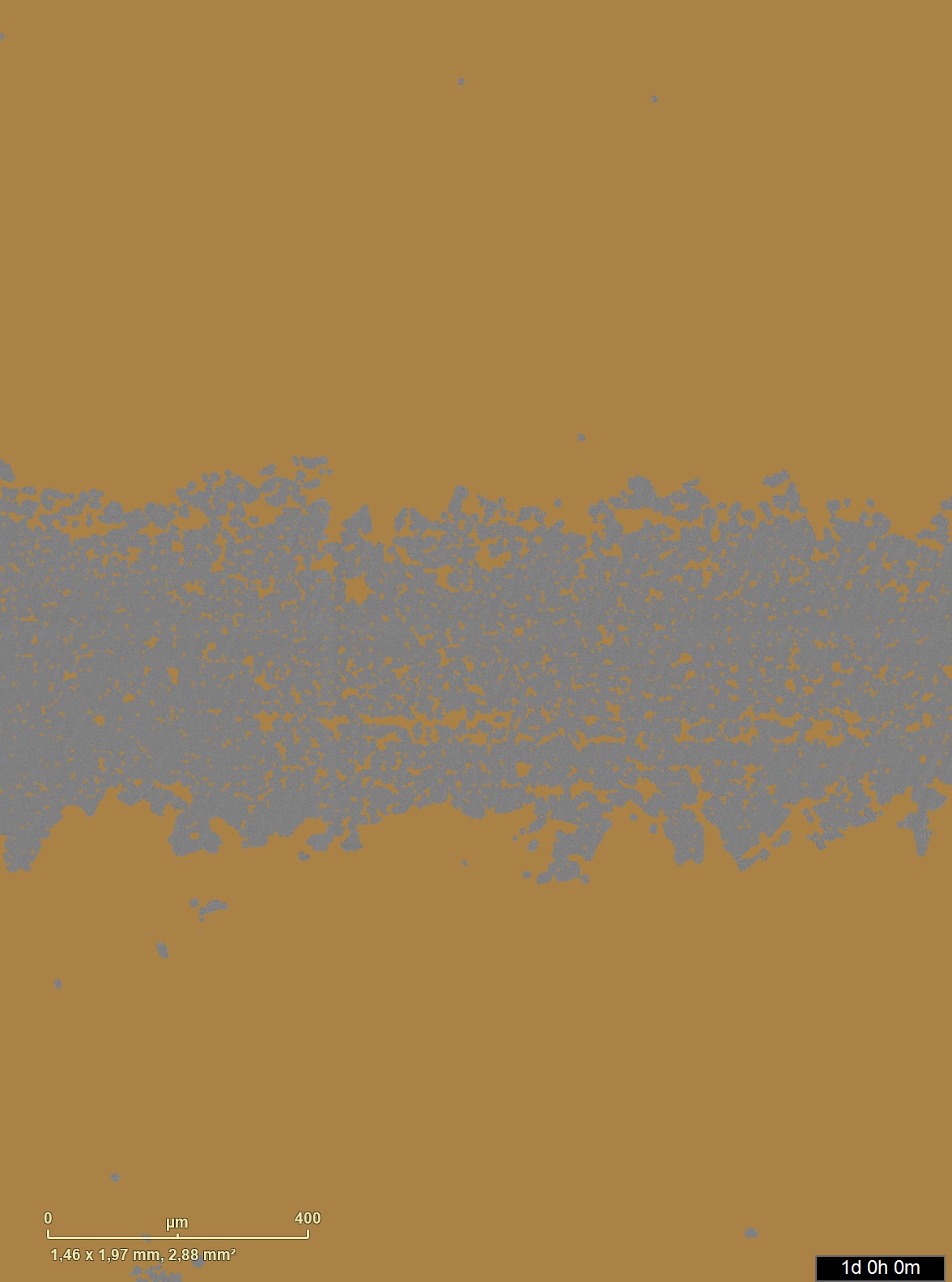

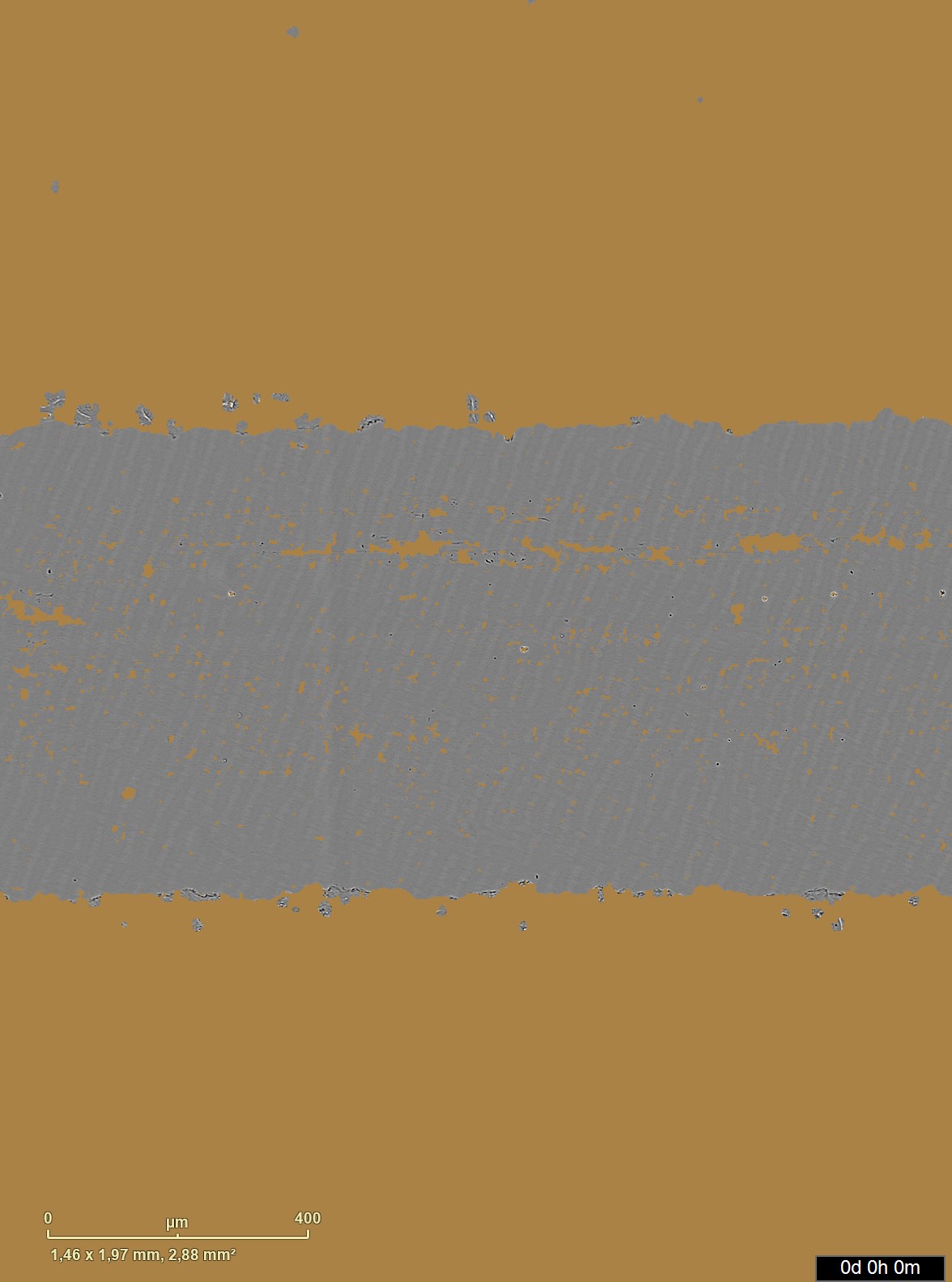

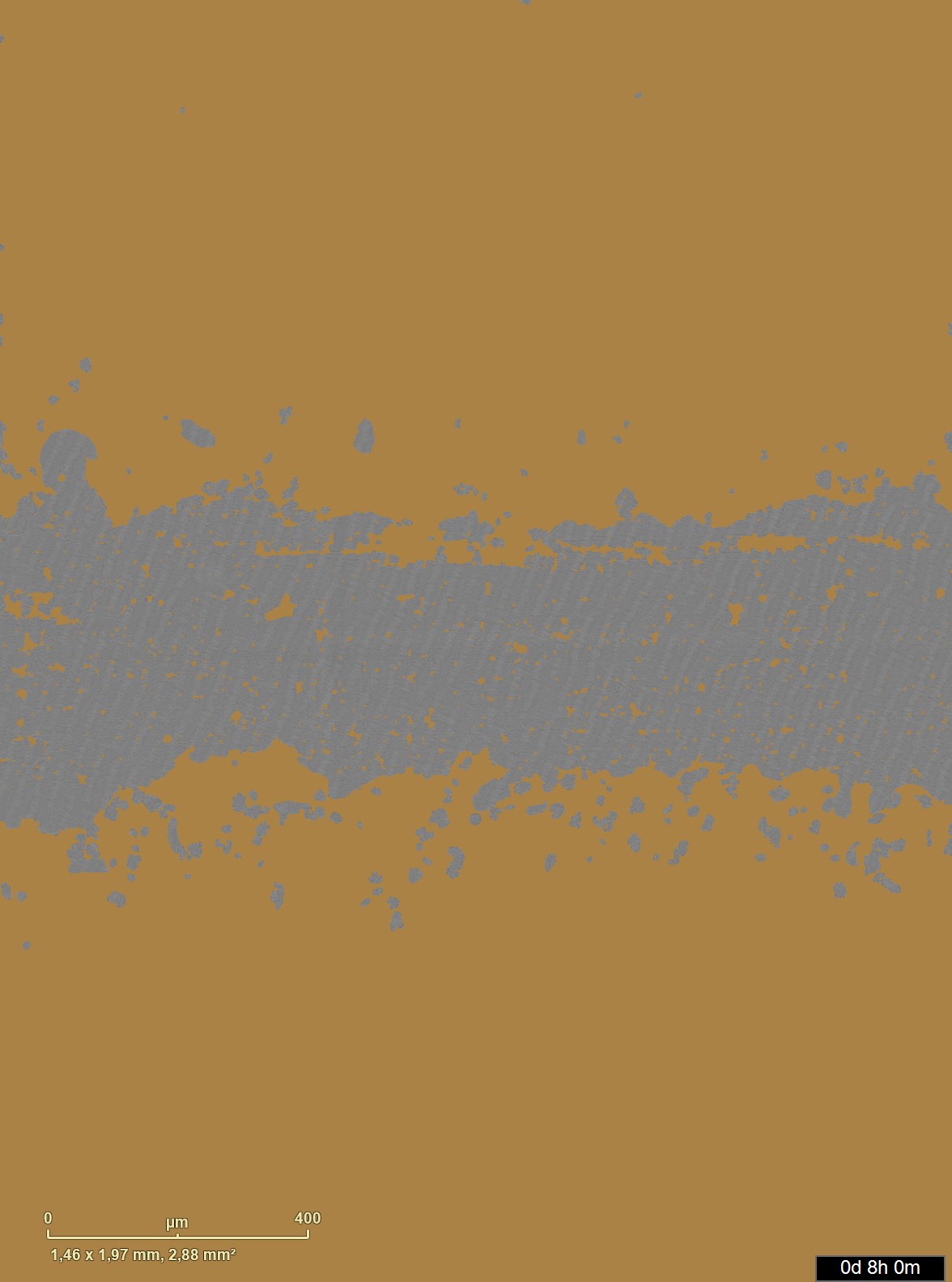

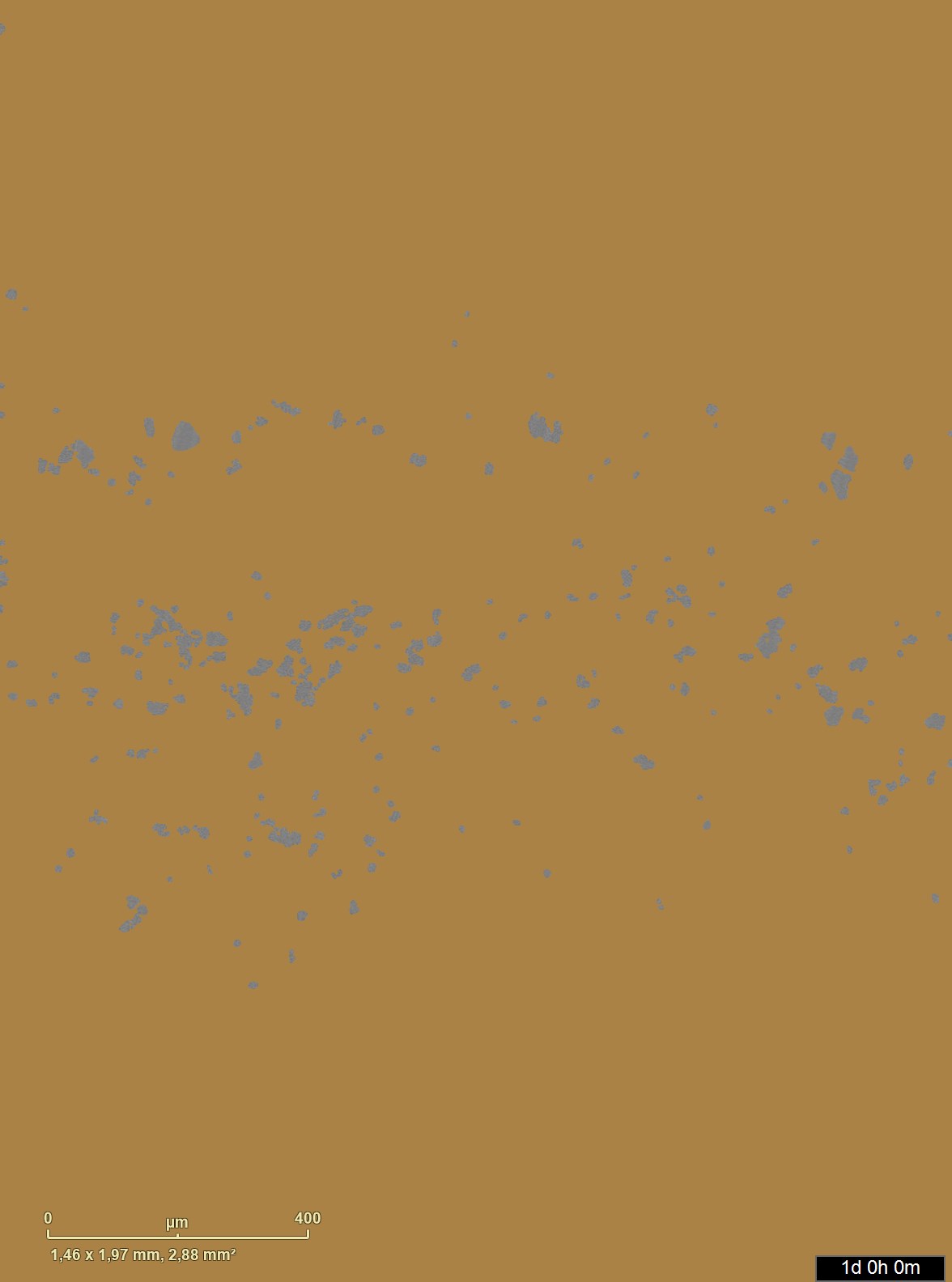

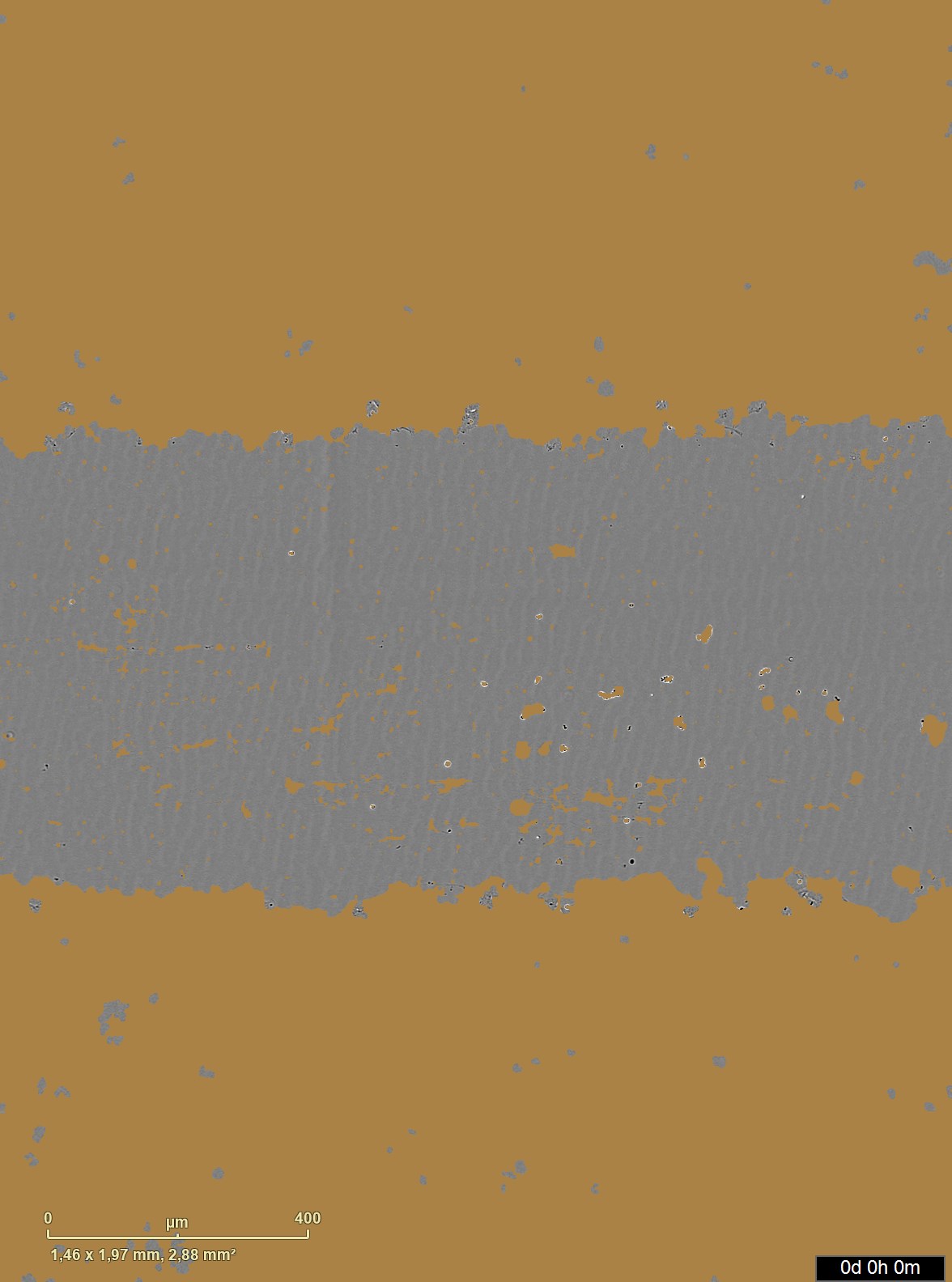

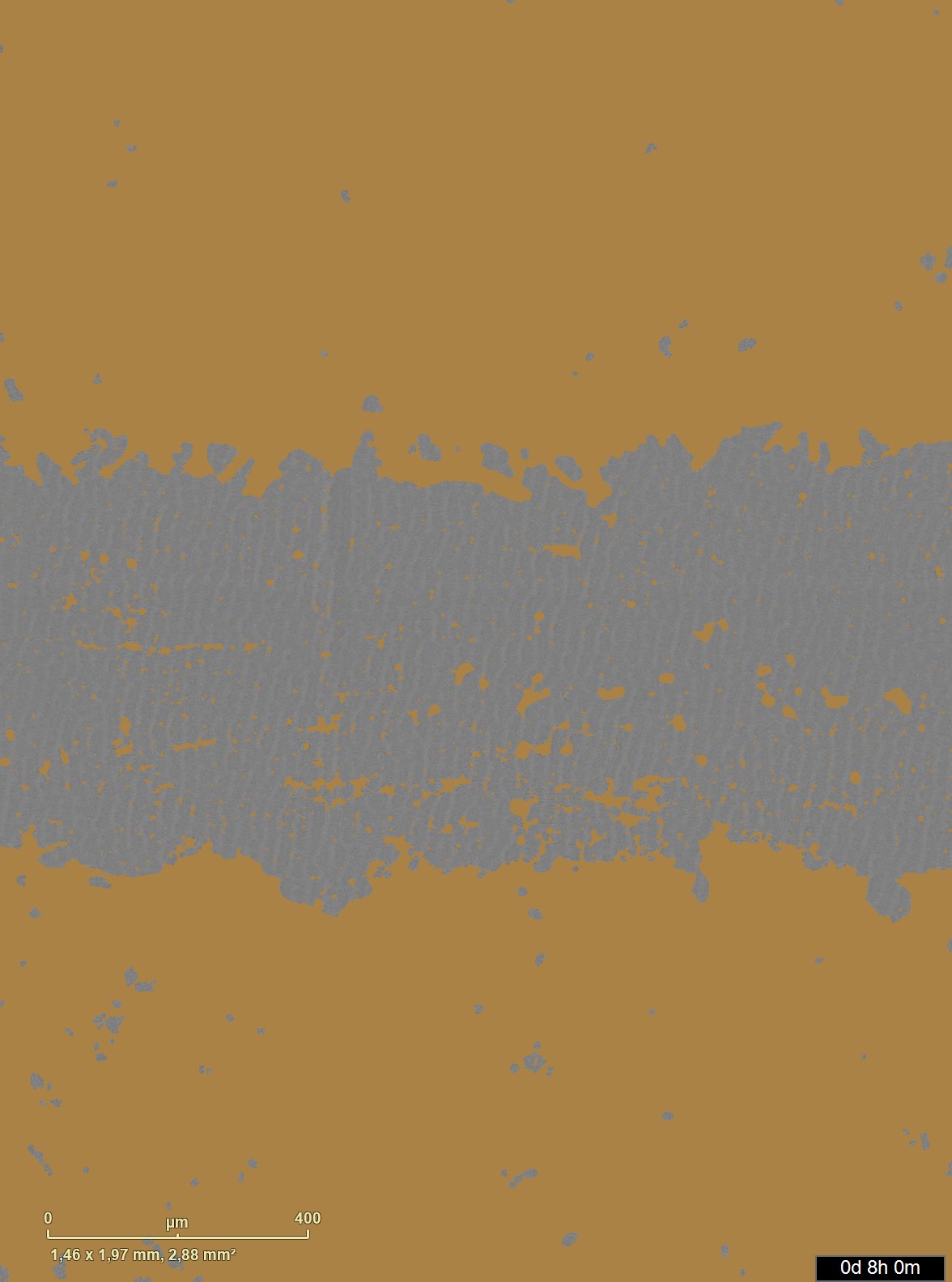

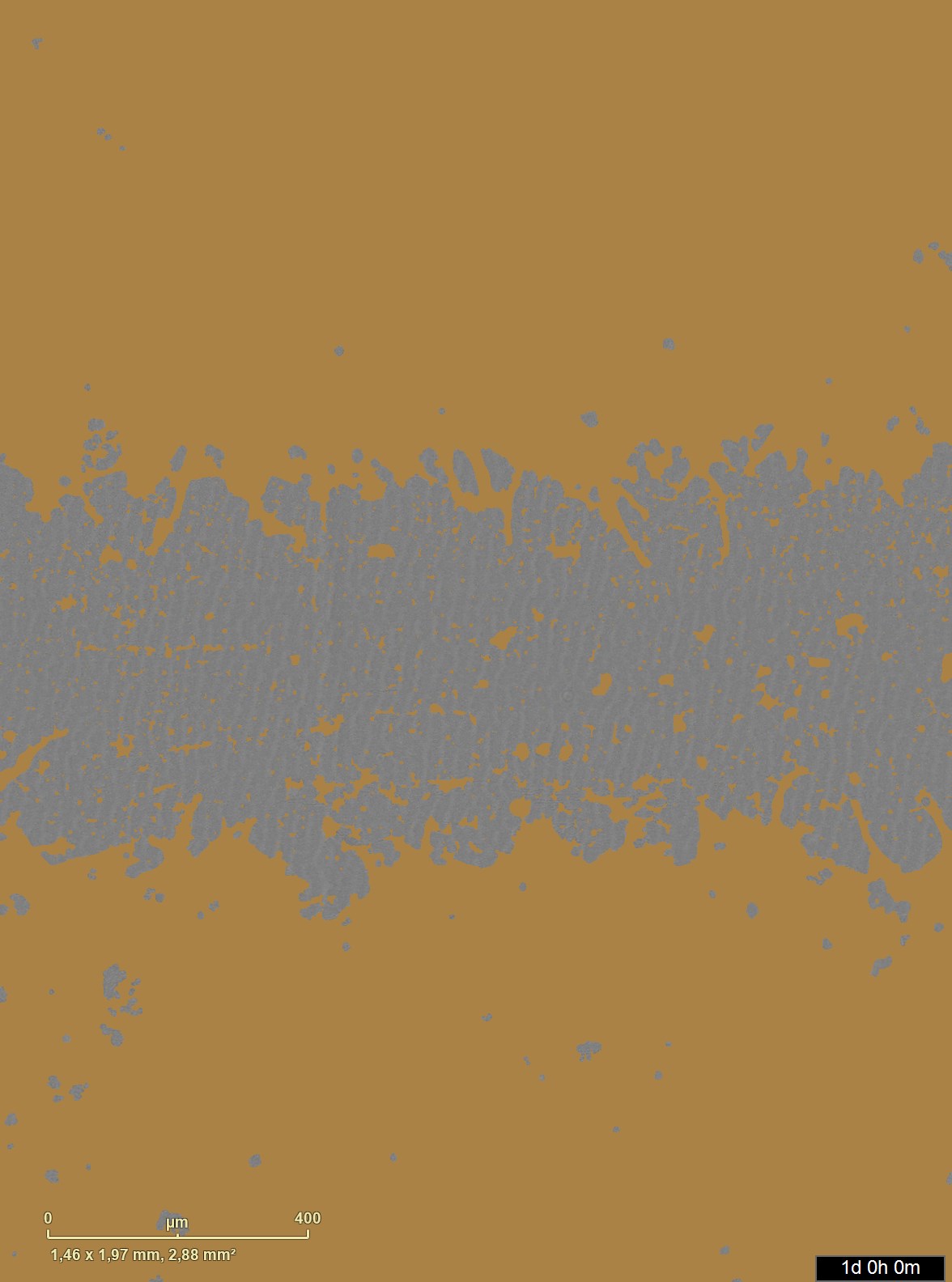

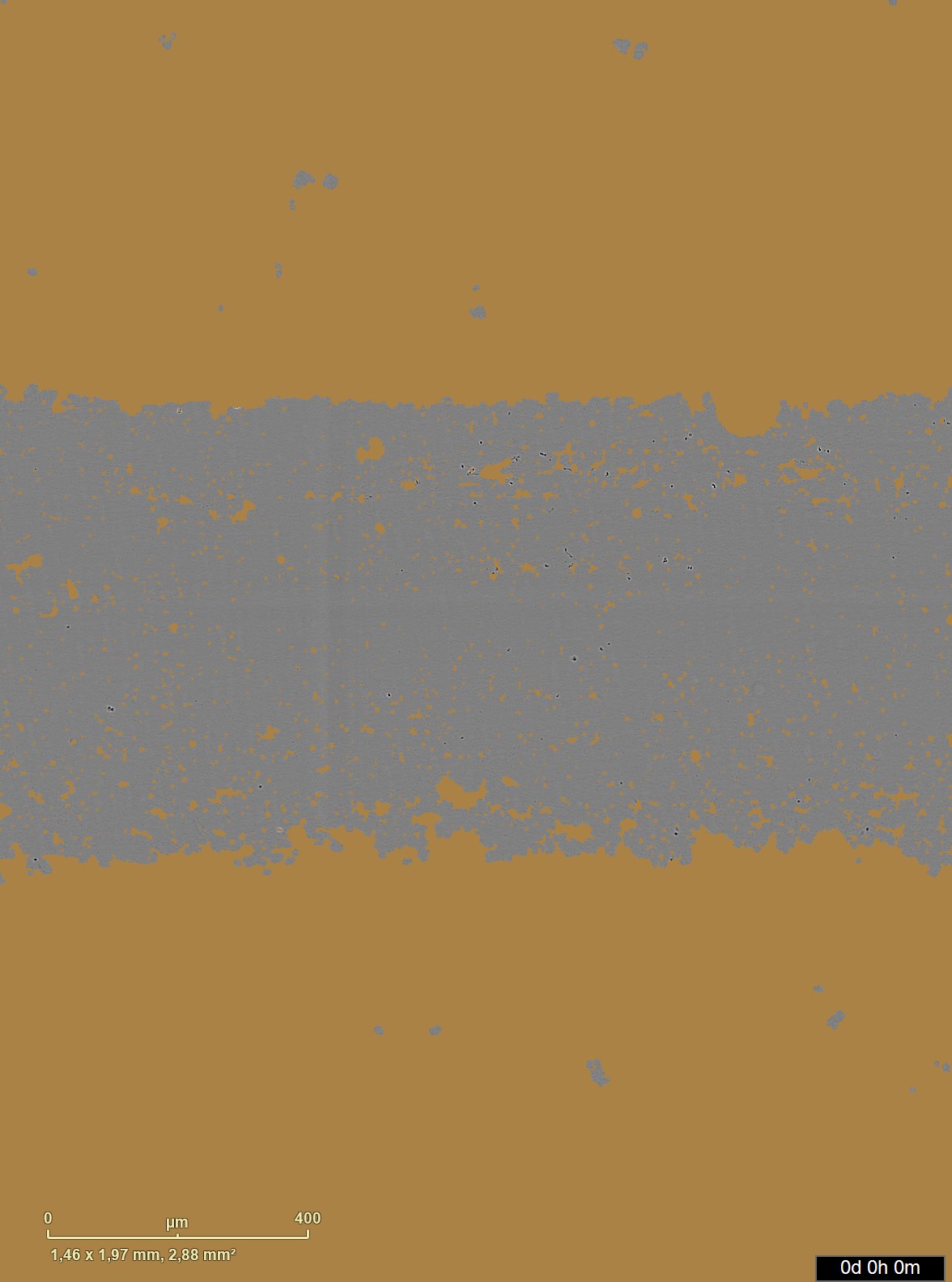

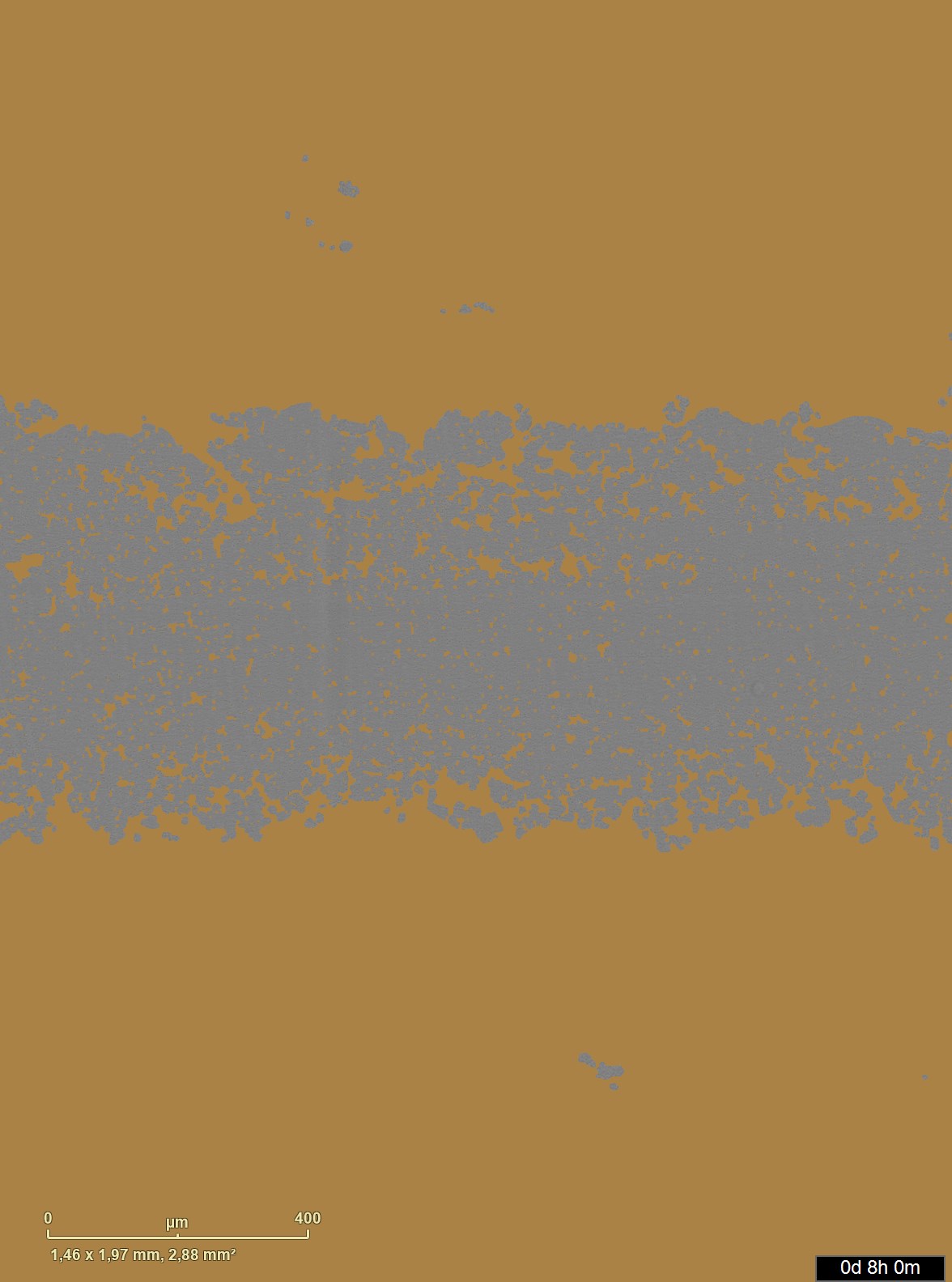

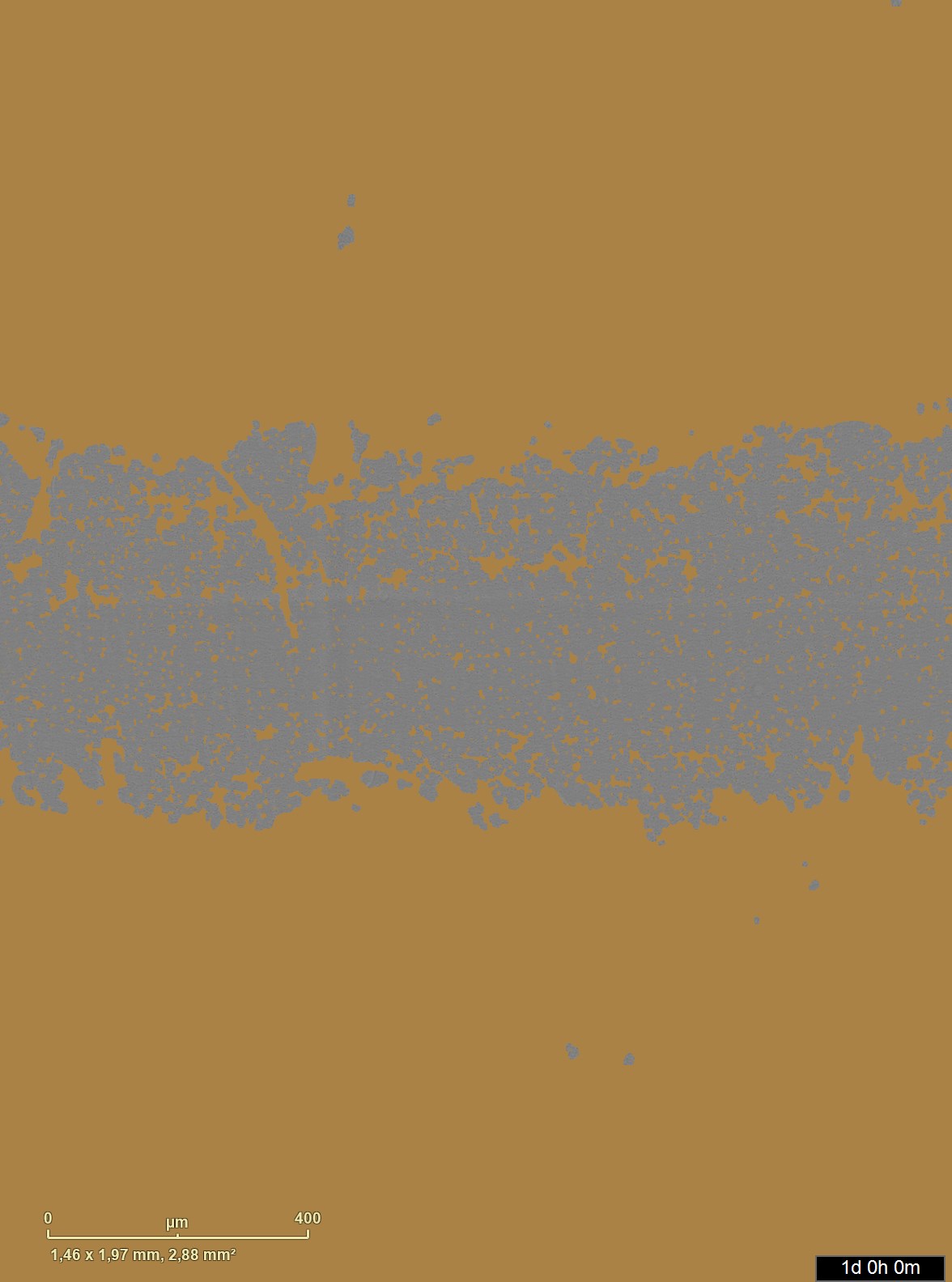

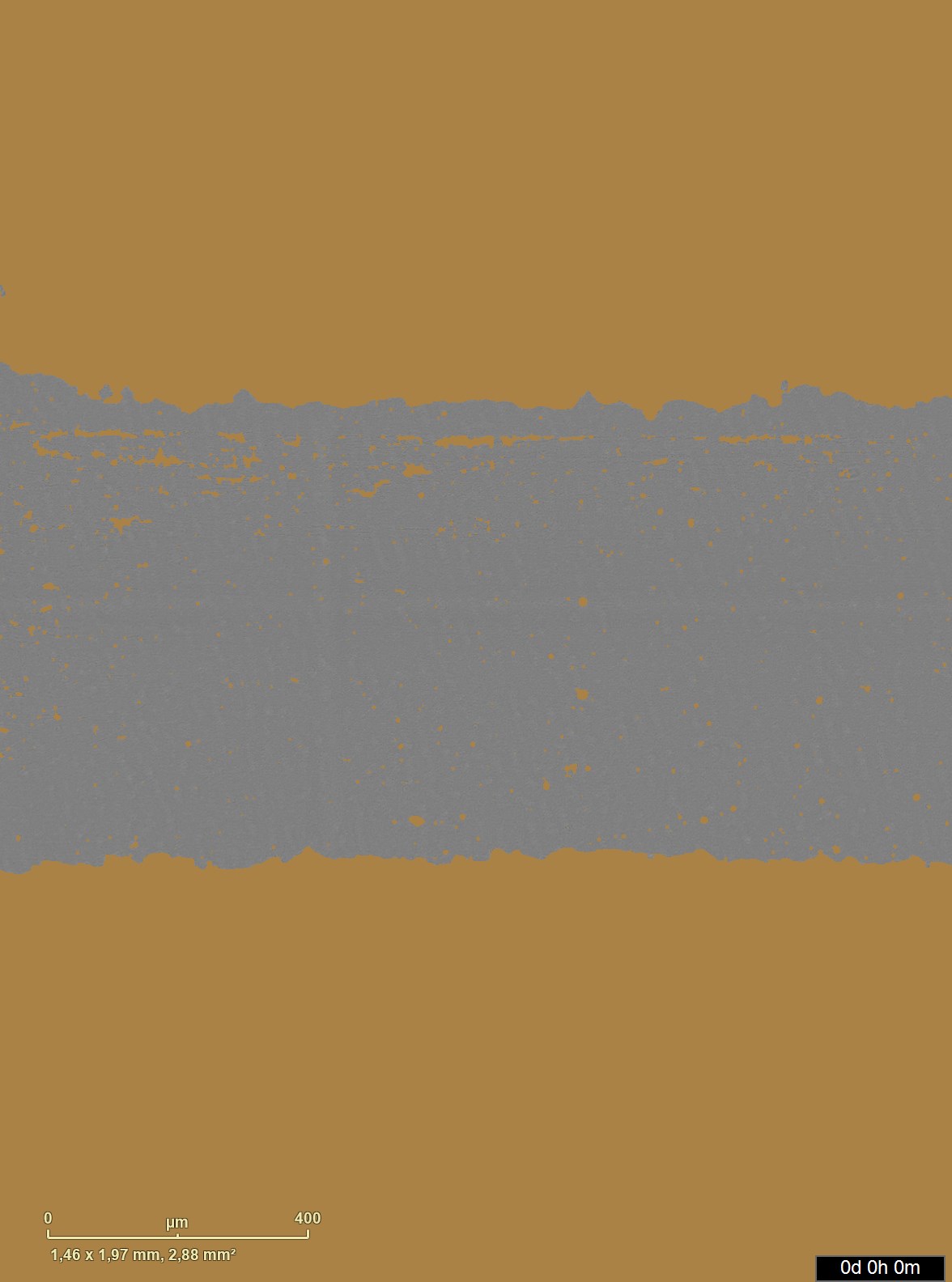

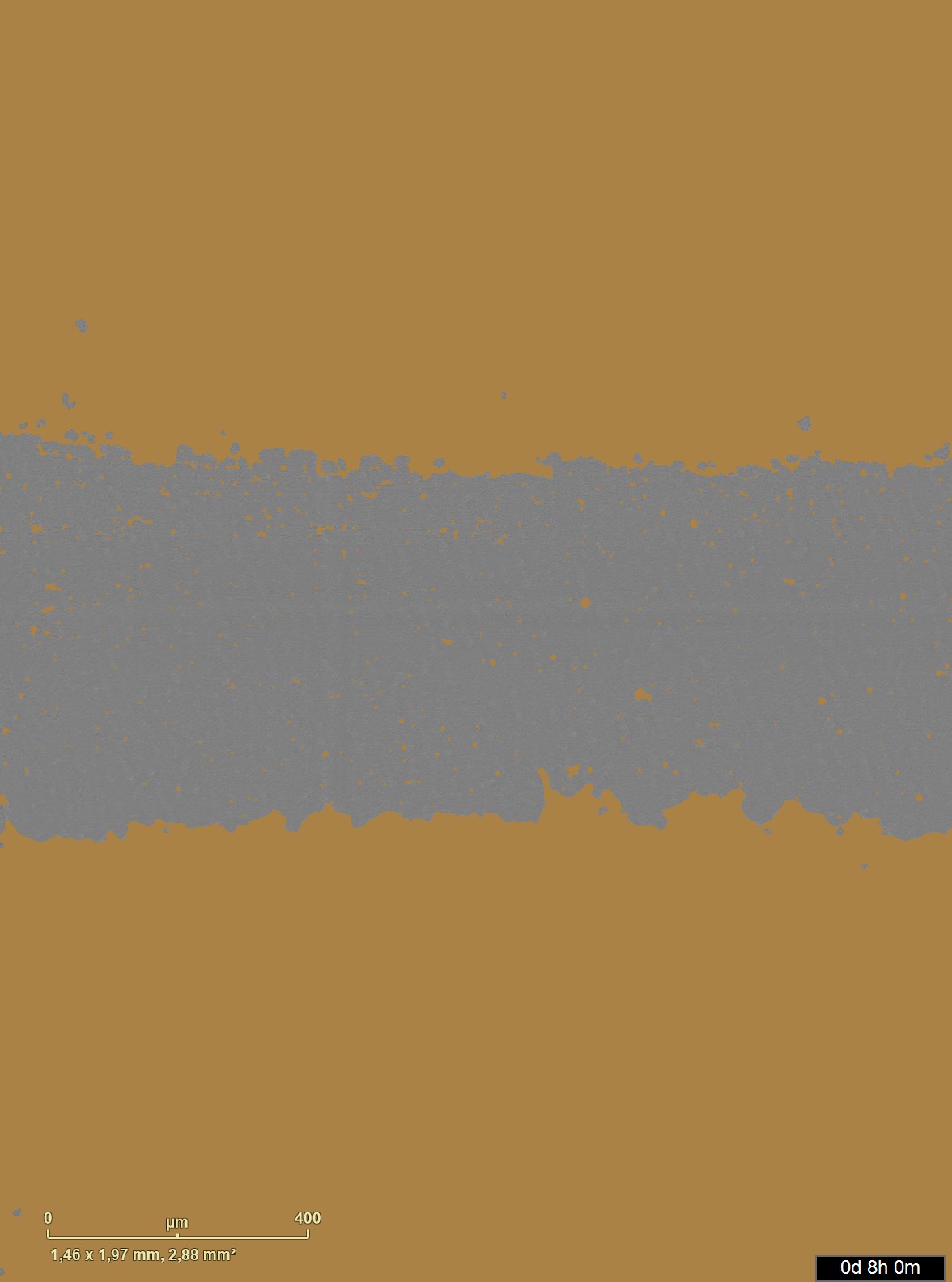

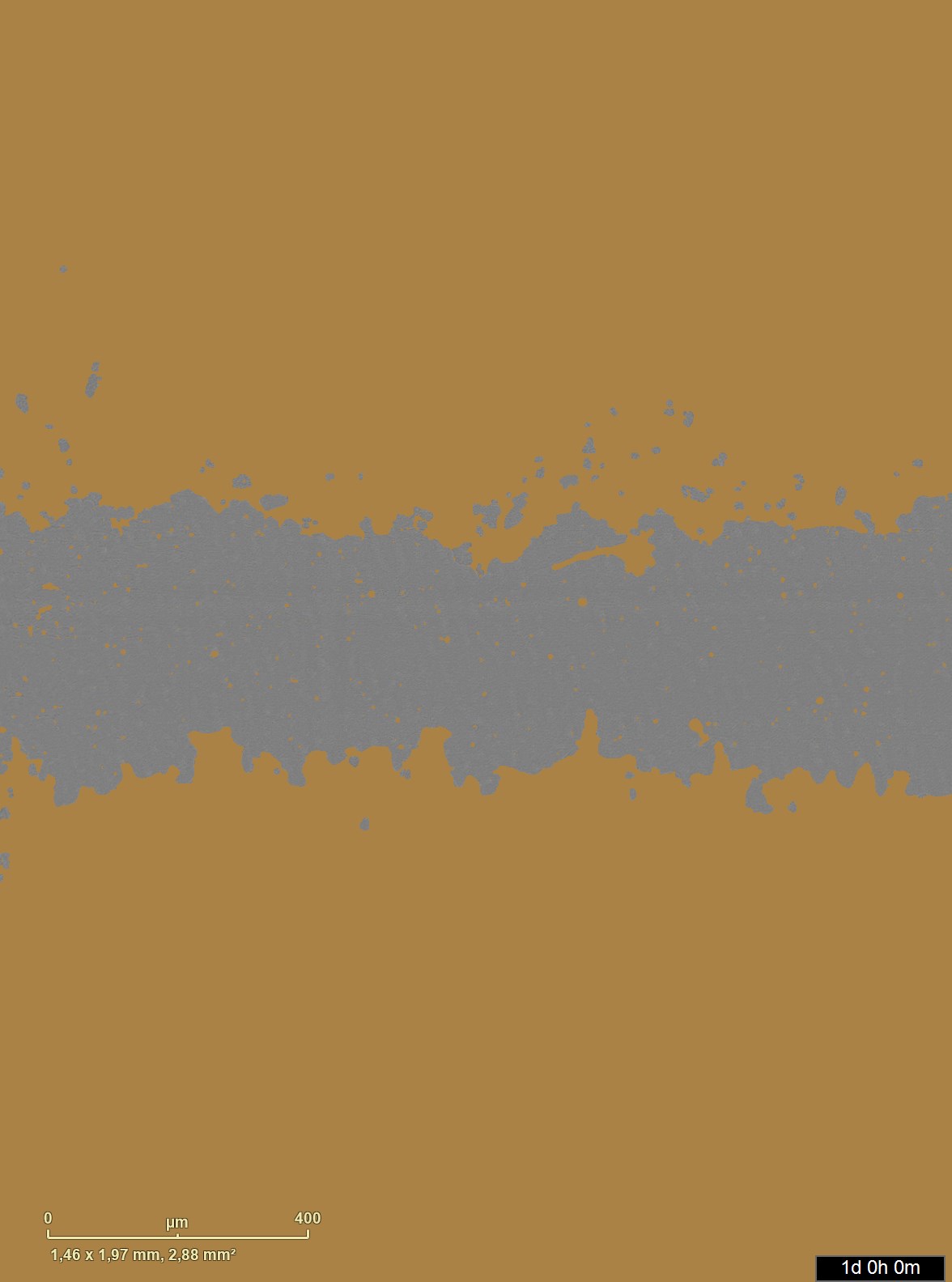


**Supplementary Figure 6.** IB105 and IB105/106 migration capacities are reduced in presence of AICAR. (A) Images of IB105, IB-106 and IB105/106 pre-treated or not with AICAR after a wound healing assay performed with the IncucyteS3. Black box represents the size of the would at time zero, the red dashed line represents the size of the wound at 8 hours and the red dotted line represents the size of the wound at 24 hours. The experiment was performed twice with twelve replicates. (B) Images obtained in (A) were processed using the wound mask from the IncucyteS3 software. IncucyteS3 software calculates the area cover by cells (yellow) and the area of the wound (grey) at each time point.
